# Use of a pathogen X tabletop exercise to assess the operational response preparedness of an emerging infectious diseases research network

**DOI:** 10.3389/fpubh.2025.1551996

**Published:** 2025-03-27

**Authors:** Rachael Lee, Jennifer Hemingway-Foday, Nefer Batsuli, L. Danielle Wagner, Aaron Macoubray, Robert F. Garry, Christine K. Johnson, Kathryn A. Hanley, Nikos Vasilakis, Souleymane Mboup, Hongying Li, Cecilia A. Sánchez, Peter M. Rabinowitz, Robert F. Breiman, Nathan Vandergrift, Eric J. Earley, Hilary Bouton-Verville, E. Candice Beaubien, E. Megan Davidson, Gretchen Van Vliet, Sara Woodson, M. Anthony Moody, Gregory D. Sempowski, Richard Reithinger

**Affiliations:** ^1^RTI International, Research Triangle Park, NC, United States; ^2^Department of Microbiology and Immunology, Tulane School of Medicine, New Orleans, LA, United States; ^3^EpiCenter for Disease Dynamics, One Health Institute, School of Veterinary Medicine, University of California, Davis, Davis, CA, United States; ^4^Department of Biology, New Mexico State University, Las Cruces, NM, United States; ^5^Department of Pathology, University of Texas Medical Branch, Galveston, TX, United States; ^6^Institut de Recherche en Santé, de Surveillance Épidémiologique et de Formations, Dakar, Senegal; ^7^EcoHealth Alliance, New York, NY, United States; ^8^Department of Global Health, University of Washington School of Public Health, Seattle, WA, United States; ^9^Rollins School of Public Health, Emory University, Atlanta, GA, United States; ^10^Duke Department of Pediatrics, Duke University School of Medicine, Durham, NC, United States; ^11^National Institutes of Health, National Institute of Allergy and Infectious Diseases, Bethesda, MD, United States

**Keywords:** emerging infectious disease, tabletop exercise, preparedness and response, global health, public health

## Abstract

In mid-2020, the Centers for Research in Emerging Infectious Diseases (CREID) Network was established to address critical gaps in research expertise and capacity in emerging and re-emerging infectious diseases (EIDs). As the Network was established during the COVID-19 pandemic, most of the Network’s research centers initially focused on SARS-CoV-2 research. By the end of 2021, the Network leadership realized that it had a blind spot with regards to research centers and their sites’ overall capacities and stakeholder connections. To foster more meaningful and deeper levels of coordination and collaboration across research centers, as well as stress-test its capacity and readiness for rapid research during an EID outbreak. CREID conducted a tabletop exercise (TTX) during its Annual Partners Meeting in August 2022. Through the 2-day TTX, participants provided insight into their institutions’ resources, stakeholder relationships, and research engagement before and after an EID outbreak; additionally, technical and operational challenges and solutions with regards to a successful outbreak research response were discussed. TTX participants’ feedback was used to improve the Network’s operational research response framework and processes. Given the limited existing resources on TTX for infectious disease outbreaks, the materials developed for the TTX and reported here can serve as a reference for determining and preparing for any research institution’s role in pandemic preparedness and response research efforts.

## Introduction

Since 1980, there has been a steady increase in the number of global infectious disease outbreaks—including those due to emerging and re-emerging infectious diseases (EIDs)—at an estimated rate of increase of 6.7% annually (1). These outbreaks represent a threat to human health and global security, and are associated with changes in climate and the human-animal interface in high-risk areas, community interconnectivity, and travel and migration patterns (2–4). Global EID hotspots include forested tropical regions where interactions between humans and animals, specifically wildlife, are frequent and land-use changes are occurring (5). Recent examples of country, regional, and global EID outbreaks include Ebola in 2014–2016 (West Africa) (6) and 2018–2020 (Democratic Republic of the Congo) (7), Zika in 2015–2016 in Latin America (8), Middle East respiratory syndrome (MERS) in 2015 (South Korea) (9), Mpox in 2022 and 2024 (10), and severe acute respiratory syndrome coronavirus 2 (SARS-CoV-2) in 2020–2023 (11). Because EID outbreaks occur at unpredictable intervals and in geographically diverse locations, the current research infrastructure has limited capacity to rapidly respond to them. The recent global experiences combating Ebola, Zika, MERS, Mpox, and SARS-CoV-2 highlights the urgent need for close coordination and international research-focused partnerships during EID outbreaks.

Critical contemporaneous research necessary to inform clinical care and the public health response during EID outbreaks has been historically slow due to physical, logistic, and operational in-country barriers and challenges. Lack of coordinated and relevant capacity, expertise and infrastructure can result in significant delays in conducting critical outbreak-related research. For example, Brazil announced Zika as a public health emergency in November 2015 (8). However, the first study site for the U.S. National Institutes of Health (NIH)–funded Zika in Infants and Pregnancy study was not activated until June 2016, and the European Union–funded ZIKAlliance did not enroll its first study participant until May 2017 (12).

In response to these barriers, in 2020, the U.S. National Institute of Allergy and Infectious Diseases (NIAID) established the Centers for Research in Emerging Infectious Diseases (CREID) Network, with the goal of enhancing global pandemic research response preparedness by building a sustainable, scalable, and adaptable infrastructure for EID research before, during, and after outbreaks. The inaugural Network comprised a central Coordinating Center (CC) and 10 Research Centers (RCs), with over 115 local research sites (RSs) in more than 30 countries where EID outbreaks are most likely to occur. Through the CREID Network infrastructure (see the Network’s research collaborators and site locations)^1^, multidisciplinary teams of investigators collaborate to study EID pathogens of pandemic potential, identify critical research knowledge gaps that inhibit the development of prevention or mitigation strategies, and support critical research efforts to better understand the pathogen’s emergence, transmission, and overall outbreak evolution while performing enabling research to inform development of essential diagnostics and targets for potential medical countermeasures (i.e., vaccines and therapeutics).

Upon detection of a disease outbreak, many available human resources are applied to a public health response—prioritizing confirmation and treatment of cases as well as swift and effective control of an outbreak before it can expand to a geographic area that is too large to manage with available resources. To promote early identification of research needs and opportunities in the setting of an emerging outbreak, the CREID Network emphasizes the value of shifting capacity to coordinated outbreak research response (ORR) efforts—with an emphasis on the research component—to address key knowledge gaps of EID pathogens’ epidemiology, genetics, host interaction, and transmission dynamics. Since 2020, the CREID Network has promoted ORR efforts in more than 22 outbreaks of priority EID pathogens and pathogens of interest. These efforts have ranged from providing diagnostic and other research protocols to training in-country researchers in advanced analytical methodologies and supporting primary sample and data collection in observational cohort studies of humans, animals, and the environment.

Two years into the launch of the CREID Network, the capacities and expertise of the RCs and their network of global sites and investigators had been curated. However, there was still an opportunity to foster more meaningful and deeper levels of coordination and collaboration across this new research network. To address this need, during the CREID Network’s second annual program meeting, a tabletop exercise (TTX) based on a fictional outbreak simulation was conducted to encourage and build a collaborative Network community, share best-known practices and resource tools curated by the CC, and stress-test the CREID Network’s capacity to rapidly launch a coordinated research response for future outbreaks.

TTXs are group activities that use progressive simulated scenarios where TTX “Players” discuss and reflect on the impact of potential situations/scenarios on existing plans, procedures, and capacities (13). Through facilitated group discussions, they simulate an emergency in an informal, stress-free environment to strengthen preparedness to manage that emergency. TTXs are used to (1) develop or review a response plan; (2) familiarize Players with their roles and responsibilities; and (3) identify and solve problems through a facilitated and open discussion. TTXs have been used to prepare national and subnational government agencies, multi/bilateral organizations, and non-government institutions for emergencies such as epidemics or pandemics (14–16), floods (17, 18), and hurricanes (19). Their use to define the role of research in outbreak settings has been limited, however.

During the development of the CREID Network TTX, the cross-network planning team observed a lack of TTX resources specific to disease outbreaks, research responses, and multilateral coordination. Most existing resources were specific to public health management at a single institution engaging directly in an emergency or disaster response. This report provides an example of a TTX specific to pandemic research network preparedness and response involving international cross-institutional coordination and collaboration.

**Figure 1:**
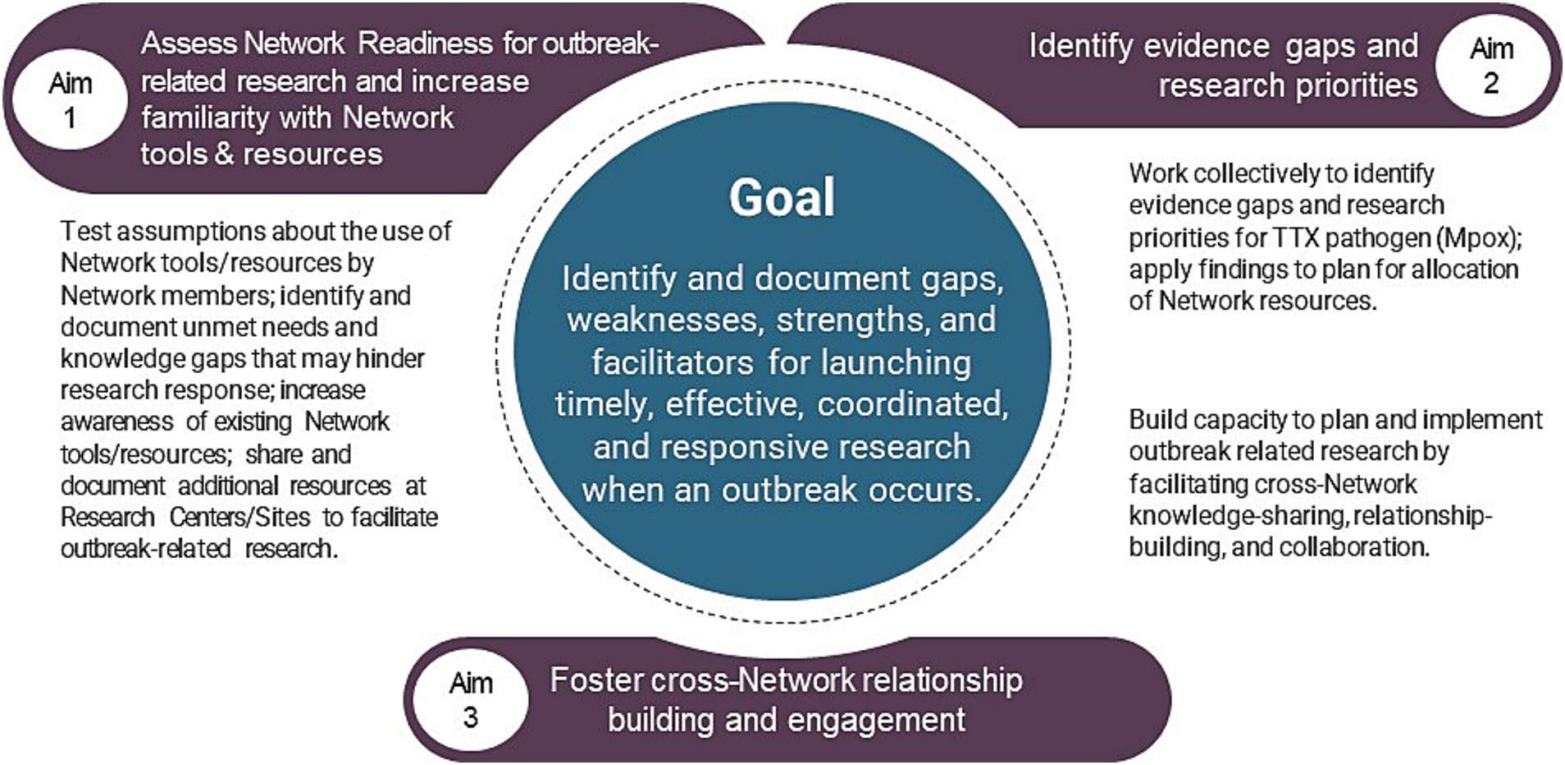
Tabletop exercise goals and objectives.

The primary outcome of the CREID TTX effort was to inform the development and adoption of a proactive framework for effective, timely, and responsive research when an EID outbreak occurs. To achieve this, the exercise was designed to help address the following aims (Figure 1): (1) assess CREID Network readiness for outbreak-related research and increase familiarity with Network tools and resources; (2) identify generalizable evidence gaps and research priorities; and (3) foster cross-Network relationship-building and engagement.

## Tabletop exercise approach and deployment

The CREID Network’s TTX organization consisted of three phases, as detailed in Table 1: (1) a Strategic Planning Phase, where a cross-network TTX team was formed; (2) a Deployment Phase; and (3) an Analysis and Dissemination Phase to produce an After-Action Report to the Network’s leadership, membership, and sponsors.

**Table 1:** Tabletop exercise phases.

Phase	Key tasks
I. Strategic Planning (6–8 Months)	Form a TTX team, led by the CREID CC’s ORR Working Group and participation from the CC working group leads for Laboratory Assays, Biorepository, Data Capture and Harmonization, and Capacity Building and NIAID staff. Define TTX goals/objectives, scope, structure, timeline, and evaluation metrics. Define working group-specific goals and objectives. Develop messaging to communicate with RCs/RSs about TTX expectations and facilitate engagement. Develop a cross-cutting scenario in the form of a SitRep (see Appendix D). Determine the appropriate deployment format and supportive tools/technology. Determine the composition of Facilitators, Reporters, Players, and Observers for each Working Group breakout group. Develop a Facilitator’s Manual and interactive facilitation tool (see Appendix A). Develop a reporting template to capture feedback during the exercise in a standardized format (see Appendix C).
II. Deployment (1 Month)	Prepare for deployment: Finalize agenda for applied sessions. Train TTX Facilitators and Reporters to effectively carry out their roles and responsibilities and deploy facilitation and reporting tools. Plan for logistical needs (e.g., room setup, audio-visual support). Present Network-wide outbreak scenario at annual program meeting general session. Implement applied sessions in five concurrent breakout groups. Conduct hotwash at the end of applied sessions to rapidly document key takeaways and action items. Share hotwash report from each breakout group at general session.
III. Analysis and Dissemination (4–6 Months)	Compile and analyze data from applied session notes, hotwash reports, and evaluation form. Draft report and circulate for review and comments. Finalize report. Disseminate report via Network communications channels and Private Portal (Resources Library).

CC, Coordinating Center; CREID, Centers for Research in Emerging Infectious Disease; NIAID, National Institute of Allergy and Infectious Diseases; ORR, Outbreak research response; TTX, Tabletop exercise.

A core planning team from the CREID CC and NIAID (Network sponsor) convened 9 months in advance of the TTX to define the TTX’s goal, aims, scope, structure, timeline, and evaluation metrics (Appendices A–E); the latter included questions to TTX participants whether the TTX had achieved its goals and aims (see Appendices B, E). Appropriate TTX deployment format and supportive tools, and the composition of TTX breakout groups (Facilitators, Reporters, Players, and Observers) were defined and aligned with CREID Network Working Groups (i.e., Laboratory Assays and Biorepository, Capacity Strengthening, ORR) (Table 2). The core team also developed a Facilitator’s Manual (Appendix A), used an interactive hybrid-amenable facilitation tool to steer the discussion and collect data on TTX evaluation questions (Menti®; Appendix B), and developed a reporting template (Appendix C) to capture standardized feedback during the simulation/exercise. Facilitators and Reporters for the TTX were academic research and faculty volunteers from the CREID CC and RCs, and were all trained by the core planning team to effectively carry out their roles and responsibilities and deploy facilitation and reporting tools when the TTX was conducted. Roles of TTX facilitators, reporters, players, support staff and observers are described in the Facilitator Manual (Appendix A).

**Table 2:** Composition of assigned tabletop exercise breakout groups.

CREID network working group	# of Facilitators	# of Reporters	Total # of players (in person/virtual)	# NIAID /U.S. Government observers (in person/virtual)
Biorepository Collaboration and Quality + Data Capture and Harmonization	3	1	83 (31/52)	10 (3/7)
Lab Assays	2	1	65 (23/42)	8 (1/7)
Outbreak Research Response (Group A)	2	1	30 (30/0)	0
Outbreak Research Response (Group B)	2	1	70 (70/0)	15 (15/0)
Outbreak Research Response (Group C)	2	2	111 (0/111)	30 (0/30)

CREID, Centers for Research in Emerging Infectious Disease; NIAID, National Institute of Allergy and Infectious Diseases.

The TTX was conducted at the NIAID hosted CREID 2022 Annual Program Meeting (September 2022, Towson, MD, USA) as two 2-h hybrid format sessions, with five concurrent breakout groups. There were 359 TTX “Players” from all 10 CREID RCs assigned to the breakout groups aligned with their CREID Working Group membership—there were 154 in-person attendees and 205 virtual attendees. Of participants, 50% were from the U.S., 8% from Asia (i.e., Cambodia, Hong Kong, India, Jordan, Malaysia, Myanmar, Nepal, Pakistan, Sri Lanka, Thailand, Vietnam), 23% from Africa (i.e., Democratic Republic of Congo, Cameroon, Ethiopia, Kenya, Liberia, Nigeria, Senegal, Sierra Leone, South Africa, Uganda), 5% from Europe (i.e., Belgium, France, Germany, Norway, Switzerland, United Kingdom) and 13% from Latin America (i.e., Argentina, Brazil, Ecuador, Nicaragua, Panama, Peru). Representatives from NIAID and other U.S. government agencies (i.e., Centers for Disease Control and Prevention; U.S. Agency for International Development) were Observers (Table 2).

**Figure 2:**
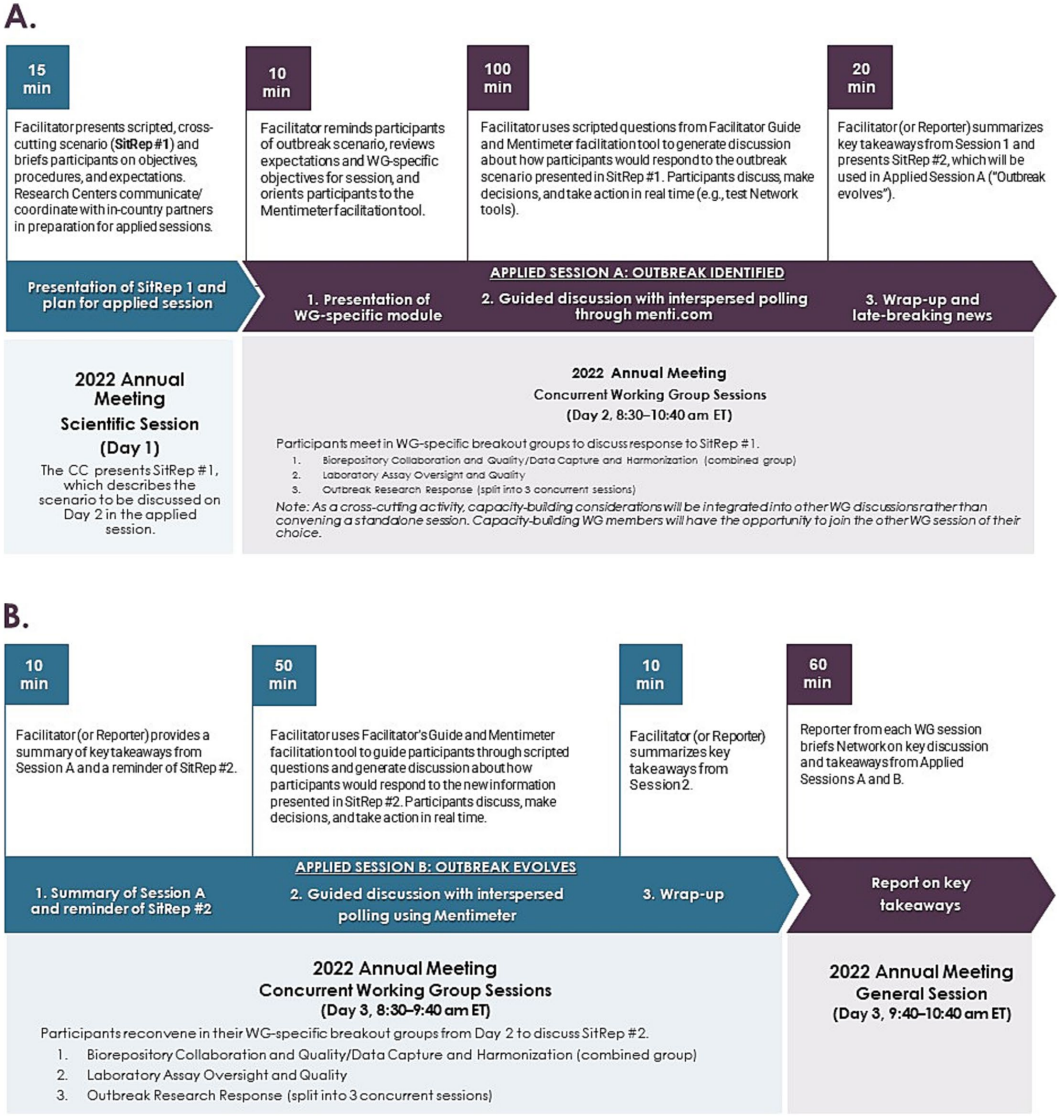
Timeline/flow of CREID network TTX activities **(A)** Days 1 and 2, **(B)** Day 3.

The TTX was based on two sequentially released complementary scenarios, structured as Situation Reports (SitReps) (Appendix D), for a fictional outbreak of an unknown Pathogen X (modeled after Mpox). The first SitRep (#1) mimicked a typical disease outbreak report, where the outbreak was identified and clinical, epidemiological, and other information shared was incomplete. The second SitRep (#2) provided more complete clinical, epidemiological, and other information, including answers to questions that arose from the SitRep#1. In each session, Players were separated into their breakout groups to discuss responses to the SitReps. At the end of each session, Facilitators or Reporters summarized key takeaways and shared a breakout group summary report at a final all-hands session. More comprehensive descriptions of the TTX activities and two applied 2-h sessions (Days 2 and 3 of the meeting) are shown in Figure 2.

Responses to TTX evaluation questionnaires, data from applied session notes, and session summary reports were included in an After-Action Report (Appendix E) summarizing key findings and follow-up action steps from the TTX. Findings were presented to the CREID Network Steering Committee and used to update and streamline the Network ORR operational framework with enhanced communication materials, roles and responsibilities matrices, and real-time tracking and reporting of Network outbreak activity monitoring and engagement.

## Observations, limitations, and recommendations

Why and how is this TTX, conducted in August 2022, of relevance almost 3 years later, and what were the most enduring lessons learned that are of value to all research centers and sites across the Network? The goal of the TTX was to foster more meaningful and deeper levels of coordination and collaboration across a new academic research network, as well as—due to the Network’s focus on EIDs—to stress-test the CREID Network’s capacity to rapidly launch a coordinated research response for future large-scale disease outbreaks. In this regard we believe that the goal is timeless, and we believe that the TTX approach described here would be of interest to a wide audience, whether affiliated with academic research networks like CREID, or to other stakeholders with an interest in EID outbreaks or health crises, such as ministries of health, multilateral/bilateral policymakers and donors, or the private sector.

### Aim 1: Assess CREID network readiness for outbreak research response and member familiarity with network tools and resources

When discussing SitRep#1 and SitRep#2, TTX Players reviewed factors and variables that could challenge ORR efforts. They identified two key components that could be strengthened or improved to ensure effective and expeditious ORR: (1) strong internal and external partnerships and (2) familiarity with CREID Network resources designed to support ORR efforts.

Building and leveraging partnerships with external stakeholders (i.e., in-country ministries of health and other host government officials, academic research institutions, non-governmental organizations, multilateral/bilateral policy makers and donors, and private sector) in CREID-focus countries was a crucial factor highlighted by participants to foster an effective environment for collaboration in ORR. TTX Players acknowledged that it takes time to cultivate productive relationships and establish buy-in with in-country stakeholders for outbreak-related research, even in longstanding partnerships where these were to exist. As such, there was strong agreement that in-country relationship-building should be viewed as a foundational activity that begins well in advance of any EID outbreak. Specifically, the government ministries relevant to the One Health sectors of Health, Agriculture/Livestock, and Environment were consistently cited as the most important in-country relationships to cultivate. As noted by TTX Players, strong relationships with these ministries help ensure that CREID researchers are “at the table when an outbreak occurs, and the public health response is launched.” TTX Players further emphasized the importance of ensuring that in-country stakeholders are well-informed of the Network’s mission and capabilities. Additionally, TTX Players consistently cited the importance of ongoing efforts to build relationships within and between Network RCs and RSs. It was noted that creating these intra-Network connections before an outbreak will better position the Network to initiate a prompt and coordinated response when an outbreak does occur.

To assess the level of familiarity with processes and resources developed specifically for the Network, Players were asked about their familiarity with various resources that the CREID CC developed for the RCs and RSs to facilitate their CREID-supported research activities. Examples of available tools and resources include outbreak notification forms (i.e., to notify the Network of an outbreak of known or unknown etiology in a country where CREID operates), Network support request forms (e.g., for sharing of pathogen-specific assays and protocols), and pathogen factsheets (i.e., summary reviews of knowledge and research gaps of selected pathogens). However, familiarity with these tools among RCs/RSs seemed to vary (Table 3). This variable familiarity with available tools alerted the CC that a review of the tools, coupled with a renewed effort to increase tool awareness among Network RCs, may be necessary. Players also confirmed the need for additional resources that were under development at the time, such as country institutional maps and a virtual repository of Network-wide available biological reagents and specimens. New resources were also suggested, including an RS capacity directory, general human subjects research protocol templates with boilerplate language, best practices for handling and transporting samples and isolates, directory of in-country partners and stakeholders that may request assistance in the event of an outbreak, universal assay platforms, viral family-level surveillance/diagnostic reagents and kits (possibly combined in a “outbreak suitcase”), support for data analytics, guidance on how CREID RCs/RSs can be officially recognized by country Ministries of Health and other government entities, and guidance on immediate and unified communication about the Network with other stakeholders.

**Table 3:** Network member familiarity with select CREID tools and resources.

CREID tool/resource	# of Members with familiarity (%)*
Network Inventory	40 (69%)
Laboratory Assay Protocols Dashboard	39 (67%)
Research Site Capacity Dashboard	32 (55%)
Outbreak Research Response Resources	31 (53%)
Research Studies Inventory	30 (52%)
Research Center and Site Map	27 (47%)
General Directory	23 (40%)
CREID Resource Library	14 (24%)
Microsoft Teams Collaborative Space	4 (7%)

*N = 58. CREID, Centers for Research in Emerging Infectious Disease.

### Aim 2: Identify EID evidence gaps and network research priorities

The TTX’s simulated Pathogen X outbreak was based on Mpox, with the goal of leveraging the TTX to facilitate development of a general roadmap for future Network engagement in critical research during a global multi-country outbreak. On Day 2, TTX Players were informed that Pathogen X was Mpox and were asked to consider potential evidence gaps and priorities for Mpox-related research. The Network could then use this information for strategic planning and resource allocation. However, the 70-min time frame that was allocated for the Applied Session B (Figure 2) did not allow for in-depth and exhaustive discussion. Additionally, although longer sessions during the annual meeting were not feasible due to Players joining from multiple time zones and having competing priorities, they should be considered in future TTXs. Instead, additional discussions continued outside of the TTX scope as part of ongoing Network ORR Working Group activities.

Although the intended goal for this aim was not achieved, the collaborative discussions generated important general recommendations to better position the Network to engage in future pathogen-agnostic ORR activities. This included the development of a list of critical research questions that should be considered for all EID outbreaks (Table 4). Network leadership uses this list to plan for research activities that could be conducted during inter-outbreak periods or rapidly launched at the start of an outbreak, including identification of facilitating resources and collaborative partnerships both within and outside the Network.

**Table 4:** TTX-generated research questions relevant to any outbreak to rapidly prepare research response activities.

Epidemiology
What are the ecological factors driving the outbreak?
What type of granular geospatial information can help better define outbreak dynamics? Relationships between location, environmental factors, and health outcomes; Geospatial mapping; Geographic spread.
Are there common animal species? What animal species are people interacting with?
What is the animal ecology relative to outbreaks (e.g., seasonality)?
What is the asymptomatic infection rate?
What are the population demographics?
How can we use predictive modeling to understand where the outbreak will go next?
What is the temporal nature of the outbreak?
What is the seroprevalence?
What are the bushmeat supply routes and transportation networks?
Genetics
Phylogeographic data, R_0_?
What is the genetic sequence of the pathogen?
What are host genetic factors?
What are differences in clinical phenotypes?
What is pathogenicity of different clades?
What is needed to define clades and sequence variation?
Are there other surveillance data that we could tap into (e.g., wastewater)?
What are the viral sequelae and long-term effects?
Host Dynamics
What is the animal reservoir?
Is the infection rate increasing in the animal reservoir?
How has the animal/host range of the virus changed? Is there a functional genomic change at play?
What is the origin of the pathogen and when/how did spillover into animals/humans occur?
What are the host factors/comorbidities?
Transmission Dynamics
What is the mode of transmission and course of infection?
What modes of transmission can we predict based on other outbreaks?
Symptomatic vs. asymptomatic infection (viral load/transmission)?
What are the transmission dynamics in both human and animal cohorts?
What role does long-term immunity play?
Is transmission zoonotic or human-to-human, or both?
Medical Countermeasures
What are best ways to detect the cases accurately?
What are appropriate sample-collection procedures?
What is the specificity of available tests? Is there any cross-reactivity with other pathogens?
What antiviral drug screening exists?
What antivirals, other therapeutics, repurpose licensed vaccines are needed?
Discovery and design of therapeutic antibodies?
Data/Analytics
What is the metadata structure?
What data would be needed for predictive modeling?
What is the analysis capacity of sequence data onsite?

### Aim 3: Foster cross-network relationship-building and engagement

Given the COVID-19 pandemic, the CREID 2022 Annual Program Meeting was the first opportunity for many Network members to connect with colleagues and collaborators from other RCs in person. Although most respondents said they did not personally know breakout group participants from other CREID RCs, all respondents reported they felt more comfortable interacting with CREID members from different RCs after the TTX—a change in behavior that likely was facilitated by CREID members meeting and actively interacting with one or more members of other RCs during the TTX.

Of 37 respondents, 84% (*n* = 31) said that they had learned something new about another RC or RS during the TTX, including their geographic presence, the scope of their research within and outside of the CREID Network, their linkages with government counterparts and other stakeholders, and general technical and operational capabilities. Similarly, two respondents specifically mentioned that they had been previously unaware of how some RCs had very close ties with Ministries of Health in various countries and how involved they were in their respective countries’ public health emergency response efforts.

### Limitations, challenges, and recommendations

Several limitations were highlighted in the scope and application of this TTX. A major limitation of the exercise was that, as an exercise, it could not replicate the setting of an occurring EID outbreak. Sense of urgency, unanticipated circumstances, competing priorities, and politics are just some of the elements that are difficult to replicate in a simulation. Additionally, although an in-depth and exhaustive discussion was planned, the 70-min time frame that was allocated for SitRep#2 did not allow for it. Additionally, due to the hybrid nature of this TTX, with Players joining from multiple time zones, and competing priorities during the annual program meeting, longer sessions were not feasible, but should be considered in future TTX. Convening a 2–3 day TTX on its own may be costly, particularly for a research network spanning more than 30 countries—therefore, to minimize costs, the TTX was conducted during the already scheduled CREID Annual Partners Meeting.

After each TTX session, the Facilitators or Reporters of each breakout group summarized key takeaways, which were presented to all TTX participants at a final all-hands general plenary session at the annual program meeting. The feedback across all sessions informed key challenges to effective ORR and international collaborative research. The TTX highlighted several key technical and operational challenges faced by researchers engaged in ORR efforts. First, Biosafety Level (BSL) 3 and 4 and U.S. Select Agent requirements, as well as cold chain requirements dictate sample storage and processing locations, posing significant logistic challenges to conduct timely outbreak research. Second, limited data sharing and inadequate financial and human resources hamper pivoting from direct outbreak engagement (i.e., the public health response) to ORR. Third, there is a notable lack of member awareness about the capacities of other RCs, their RSs, and external stakeholder connections, which hinders timely and effective mobilization of existing capacities and resources across the Network. Fourth, the Network also faces challenges with proactive coordination and limited in-person interactions across its large global footprint, which often leads to reactive—rather than strategic—efforts being implemented. Table 5 summarizes key challenges, many Player recommendations, and high-level actions taken by Network leadership to address the recommendations. The rationale behind these recommendations is to help the CREID Network continue to grow and evolve to be a capable, experienced, and trusted EID pandemic preparedness and response stakeholder that can not only conduct critical outbreak-related or adjacent research to improve general knowledge on specific EID pathogens, but also complement and/or support an overarching public health response. Having internal and external linkages and relationships established prior to an outbreak may also facilitate the planning and implementation of ORR efforts. Additionally, in the event of an outbreak, Players noted that clear roles and responsibilities of engaged RCs and RSs in any ORR effort should be defined at the outset, to avoid overlapping efforts, misrepresentation, and miscommunication.

**Table 5:** Challenges, recommendations, and actions taken following TTX.

	Recommendations	Actions taken
BSL-3 and 4 requirements and select agent designations dictate where samples can be processed and stored	Discuss with NIAID/DMID how CREID RSs can build the country or regional infrastructure to handle samples/pathogens at BSL-3 and 4 and comply with U.S. Select Agent requirements, and thereby avoid international shipping of samples.	RCs explored RS infrastructure needs for ongoing and future EID research.
Cold chain requirements	Discuss with NIAID/DMID how to improve RSs cold chain shipping capabilities in-country to ensure sample / specimen stability for successful testing and characterization.	
Limited data sharing among Network members	Embrace the NIH/NIAID requirement for public sharing of data assets associated with CREID-funded publications and shared protocols.	Created CREID Data Hub Developed human subject protocol templates. Increased multi-RC collaborations, including trainings, research studies, and manuscripts.
Limited financial and human capacity to shift efforts into ORR from direct outbreak engagement	Provide rapidly accessible, flexible ORR funds that RCs can utilize at the onset of an outbreak. Produce standard (“boilerplate”) human study protocols to save time to get protocol approved and launch research following outbreak.	Developed human subject protocol templates. Launched Tiger Teams during multi-RC and multi-country outbreaks.
Lack of awareness of other RC capacities and stakeholder linkages	Coordinate with RCs/RSs to collect information on capacities and stakeholder linkages. Develop tool or resources that allows sharing of this information across the Network.	Created country institutional map briefs. Launched a virtual BioDirectory of specimens and reagents. Updating the site capacity dashboard and lab assay dashboard.
Unchartered territory of Network-level coordination	Create connections within and between prior to an outbreak occurring so that resources can be mobilized when the outbreak does occur—knowing RCs/RSs infrastructure and operational/staffing capabilities, and in-country linkages with stakeholders such as MOHs and other stakeholders (see above) are critical to effectively and expediently launching research activities in the event of an outbreak. Define and delineate clear roles and responsibilities of the RCs/RSs for engagement in the ORR effort to avoid overlapping efforts, misrepresentation, and miscommunication	Developed the Network communications toolkit. Launched Tiger Teams during multi-RC and multi-country outbreaks.
Reactive rather than proactive actions related to ORR	Shift from reactive to proactive ORR by investing more time preparing for high-priority ORR and less on tracking, reporting, and responding to every outbreak.	Developed human subject protocol templates. Provided guidance for RC/RS to receive broad IRB approvals, that—when outbreak does occur—only require amendment rather *de novo* IRB application. Developed pathogen research factsheets and roadmaps.
Limited in-person engagement across large Network	Continue to hold CREID Annual Program Meetings. Ensure that the Annual Program Meeting’s agenda includes CREID-supported research activities, an overview of infrastructure and capabilities, and in-country linkages with stakeholders such as MOHs; additionally, the meeting should include discussion of relevant non-CREID collaborative research and engagement with stakeholders.	RCs co-led various working groups. RCs led development of CREID Annual Program Meeting agenda.

BSL, biosafety level; CREID, Centers for Research in Emerging Infectious Disease; DMID, Division of Microbiology and Infectious Diseases; EID, Emerging infectious disease; MOH, Ministry of Health; NIAID, National Institute of Allergy and Infectious Diseases; NIH, National Institute of Health; ORR, Outbreak research response; RC, research center; RS, Research site.

#### Post TTX: updated CREID network outbreak research response framework

The recommendations generated by the 2-day TTX laid the groundwork for the CREID CC to develop a revised and simplified CREID Network ORR Framework, as well as to develop and augment Network tools and resources to best meet the needs of the Network, NIAID, and global partners and stakeholders. Several of the tools and resources developed aligned directly with recommendations from the TTX (Table 5). The revised ORR Framework also builds on the experience and learnings from the first 3 years of CREID Network operations and comprises four key components: an outbreak dashboard, use of outbreak Tiger Teams, an outbreak toolkit, and a communications toolkit (Table 6).

**Table 6:** Key components of the CREID outbreak research response framework.

Tool/resource	Description and purpose
Outbreak Dashboard	A central, limited-access, web page with dynamic information relevant to the selected outbreak, including documents, personnel, support requests/resolution, key partners/stakeholders relevant for this outbreak, description of funding sources and amounts, and key assets related to that specific outbreak, including links to shared data and summary of accomplishments that facilitates reporting, tracking, and monitoring of all PPPOI outbreaks reported to the CC.
ORR Tiger Team	A focused multidisciplinary team with representation from the RCs/RSs, NIAID/DMID, CC, and key stakeholders with needed capabilities and expertise for a specific outbreak or family of outbreaks in more than one country to create a separate space for engaged RC/RSs to communicate, share knowledge, and find points of collaboration.
Outbreak Toolkit	
PPPOI Factsheets and Research Roadmaps	Host and transmission information, characteristics, epidemiology and burden of disease, pathogenicity, pandemic potential, laboratory requirements, CREID Network relevance and known capacity, current status of pathogen research landscape, critical challenges, needs and knowledge gaps.
Institutional Mapping Country Briefs	Key government and non-government institutions and actors that are leading and supporting country-level response to EID outbreaks, specifically with regards to ORR.
Human Subjects Protocol Templates	Broad protocols and associated consent materials that include language for specimen future use and sample and data sharing to quickly conduct pathogen/host surveillance, pathogen transmission and pathogenesis, and host immune response in a pathogen X outbreak situation.
Network Pathogen Surveillance Dashboard/Map Tool	RC/RS-level pathogen hit tool that contains summary-level data from specific assay results for a single pathogen or family that will be used to inform ORR WG activities.
Network Virtual Specimen and Reagent BioDirectory	Virtual repository of Network-wide available biological specimens and key biological reagents to rapidly identify Network members that may have materials that could be shared to promote collaboration and ORR.
Communications Toolkit	
One-Page Brochure	Document that uses common understanding to explain the mutual (global) value of the Network and the potential lifesaving benefits it may provide and explains broadly how a research network like CREID functions.
Technical Brochure	A back page will be added to the above one-page brochure with expanded descriptions of the RCs, their sites, research goals and objectives, active human and animal cohort studies, technical capabilities, stakeholders, and collaboration opportunities.
Network Talking Points	Document that provides *short, pithy, memorable, and meaningful* unifying messages to use when speaking to others, including specific ways to refer to funders, collaborators, projects, as well as a list of “do’s” and “don’ts” for speaking about the Network.
Network Overview Slide Deck	A short (3–5 slide) updated CREID Network Overview PowerPoint slide deck for general audiences with no scientific jargon.
Network Technical Overview Slide Deck	A long-format (10–15 slide) updated CREID Network Overview slide deck for technical audiences with expanded descriptions of the RCs, their RSs, pathogen focus areas, research goals and objectives, active human and animal cohort studies, key stakeholders, and technical capabilities.
Member Introductions Guidance	Document providing guidance on how individual RC investigators introduce themselves as a member of the CREID Network when engaging with global stakeholders and in-country government and non-government partners.
Funding Acknowledgement	A vetted acknowledgment statement of the NIH/NIAID funding that is critical on all communications and publications.
Network Public Website	Pre-read resources to engage with public officials, the general public, and the media. Website includes sign-up for CREID Network Newsletter and LinkedIn page (http://www.creid-network.org), as well as details of the CREID Pilot Research Program (https://creid-network.org/pilot-program).
Media Training	Semi-annual, virtual group training by the CC team and an external CC-contracted Communications Consultant, and occasional private training or practice sessions for key Network spokespersons and high-consequence engagements.

CC, Coordinating Center; CREID, Centers for Research in Emerging Infectious Disease; NIAID/DMID, National Institute of Allergy and Infectious Diseases; Division of Microbiology and Infectious Diseases; EID, Emerging infectious disease; ORR, Outbreak research response; PPPOI, Priority pathogens and pathogens of interest; RC, Research Center; RS, Research Site; WG, Working group.

Except for the Network Pathogen Surveillance Dashboard/Map Tool, all components of the CREID ORR Framework have been developed by the CC and launched on the CREID Private Web Portal to date. The ORR dashboard is live for Network members and contains detailed tracking records for EID outbreaks of relevance to the Network. For outbreaks occurring in more than one country with multiple RCs engaged, Tiger Teams were established to coordinate ORR activities within the Network. The teams are multidisciplinary teams with representation from the RCs/RSs, NIAID/DMID, and CC, that are formed to create a separate space for engaged RC/RSs to communicate, share knowledge, and find points for research collaboration in the rapidly evolving landscape that EID outbreaks typically represent. The teams focus on the research response that may be needed or could occur during an EID outbreak to answer critical pathogen knowledge gaps. The Tiger Teams are not necessarily intended to directly contribute to reduce or control incidence/spread—this is a function that sits squarely with countries’ ministries of health and public health response stakeholders. As of October 2024, four Tiger Teams have been created: Sudan ebolavirus disease in Uganda (2022), Marburg virus disease in Equatorial Guinea and Tanzania (2023), *Aedes*-borne diseases in Latin America (2023), and Oropouche virus in Latin America (2024). Key components of the outbreak toolkit have been developed, including 20+ institutional mapping briefs for countries the CREID Network operates in, a virtual specimen and reagent BioDirectory for efficient material identification and sharing among RSs, human subject protocol templates, and best practices guidance for sample and data sharing. Furthermore, research roadmaps for chikungunya and Lassa fever were developed, and those for Rift Valley fever and Crimean-Congo hemorrhagic fever are currently under finalization. The communications toolkit for the framework was completed in May 2024 and uploaded to the CREID Private Web Portal’s Resource Library. The toolkit includes an updated Network brochure, key talking points and media guidance, and standardized presentations to introduce the CREID Network to external partners and stakeholders. The revised CREID Network ORR Framework will help facilitate Network member engagement with critical external collaborators and improve central coordination within the Network by providing mechanisms to leverage cross-RC or cross-RS synergies, avoid duplication of efforts, and conduct impactful research in response to outbreaks of priority pathogens and pathogens of interest.

## Conclusion

Through the use of a TTX, the CREID Network was successful in fostering stronger cross-Network relationships and assessing network readiness for research in the setting of EID outbreaks. Researchers from various regions who had been communicating online for 2 years due to the COVID-19 pandemic were finally able to meet in person at the CREID 2022 Annual Program Meeting where the TTX was conducted. The TTX underscored the need for closer relationships with government ministries that would allow them to see the CREID Network as an available research partner as well as the need for more suitable Network tools and resources that fit the needs of the in-country research teams. Although EID pathogen-specific evidence gaps and research priorities were not specifically identified, the participants were successful in narrowing down a list of critical pathogen-agnostic research questions that would be beneficial across the Network to capture key information that might have previously been overlooked and to standardize data collection processes across collaborating RSs. The CREID CC used the TTX feedback and findings to inform a revised Network ORR Framework, which was developed and implemented in the middle of Year 3 of the Network’s 5-year performance period.

The TTX approach described in the manuscript, as well as the materials developed post-TTX could serve as a reference for similar research networks and organizations to play out various scenarios to help them prepare for and ultimately respond to an emergency, such as a large-scale disease outbreak or even pandemic. The benefit of working within a network structure such as CREID is the ability to leverage capacities at various institutions for strategic planning and efficient resource allocation. From prior major EID outbreaks, including the COVID-19 pandemic, we have seen that outbreak responses typically start out as being reactive—however, proactive and coordinated engagement is required to prevent further pathogen spread. Challenges identified and solutions discussed during and after the TTX helped the CREID Network to apply a more proactive and forward-looking approach to engage in coordinated, collaborative, and impactful global outbreak-related EID research.

## Data Availability

The original contributions presented in the study are included in the article/Supplementary material, further inquiries can be directed to the corresponding author.

## Footnotes

^1^ https://creid-network.org/
