# Supplementary material for: Use of a pathogen X tabletop exercise to assess the operational response preparedness of an emerging infectious diseases research network
Source: Front Public Health. 2025 Mar 27;13:1551996. doi: 10.3389/fpubh.2025.1551996 (PMC11983644; doi:10.3389/fpubh.2025.1551996)
Supplement: Supplementary file 1 [file Data_Sheet_1.docx]

CREID Network Tabletop Exercise (TTX): Outbreak Research Response to Pathogen X

FACILITATOR HANDBOOK

**September 21–23, 2022**

This Facilitator Handbook provides exercise Facilitators with all the necessary tools for their roles in the exercise. It contains information found in the Situation Report provided to Players and Observers and has been augmented with information necessary to conduct the exercise and facilitate valuable discussion. Therefore, under no circumstance should it be distributed to anyone outside of the TTX Planning Team.

FACILITATOR *QUICKSTART* GUIDE

EFFECTIVELY FACILITATING A TABLETOP EXERCISE (TTX)

**THE ROLE DEFINED**

Facilitators guide the roll-out of the tabletop simulation. The primary role of the facilitator is to ensure that the participant discussions remain focused on the exercise objectives and issues are explored as thoroughly as possible within the available time.

**AN EFFECTIVE FACILITATOR**

- Keeps discussions on track and drives play to meet exercise objectives.
- Controls group dynamics and manages strong personalities.
- Speaks competently and confidently without dominating the conversation.
- Has subject-matter expertise or experience.
- Has an awareness of Network plans and procedures.

 Captures key findings and discussion points

**What is a TTX?**

A TTX is a facilitated discussion of a plan in an informal, low stress environment. It is like a problem-solving or brainstorming session where participants share capabilities and solve problems as a group based on their organization’s existing plans and the determined objectives of the exercise. The success of the exercise is determined by feedback from participants and the impact the feedback has on the evaluation and revision of policies, plans, and procedures.

**Why Run a Tabletop Exercise?**

TTX build organizational capacity, help organizations evaluate their business continuity plans and identify strengths and areas for improvement. These exercises provide training and awareness to staff who have an opportunity to rehearse their roles and responsibilities during an incident. Plus, because of the fictional nature of the exercise, they are low-cost and low-stakes.

**MITIGATING EXERCISE FAILURE EARLY**

Oftentimes a failed tabletop exercise (i.e., the objectives were never addressed and/or no meaningful insight documented) can be traced to failed/poor exercise facilitation.

Ideally, the Facilitator has subject-matter knowledge, is familiar with the structure and resources of the CREID Network, and has facilitation experience; however, the ability to keep discussions on track is more important than specific subject-matter expertise. Persons asked to serve as Facilitator are *strongly* encouraged to self-assess the appropriateness of their tasking and discuss any concerns with the CREID Coordinating Center as early as possible.

**FACILITATING TABLETOP SIMULATION (DISCUSSION)**

A key Facilitator role is to (1) **encourage all participants to contribute to the discussion**; and (2) **remind them that they are discussing hypothetical situations in a no-fault environment**. Facilitators also build and maintain an environment where all the participants feel comfortable, speaking honestly, and where differences of opinion are respected. Facilitators should ensure that everyone feels included in the conversation and has an opportunity to participate. **Facilitators should not lecture or dominate the discussion—rather they should keep conversations moving.** Additionally, Facilitators may want to use an issues list or “parking lot” to document valid points that are raised by participants during the exercise but that risk taking the conversation off topic; these items can be assigned for later discussion to the appropriate persons.

**TTX NORMS & ADMINISTRATIVE CONSIDERATIONS**

Facilitators should control group dynamics by establishing norms at the start of the exercise (e.g., discourage side conversations, ensure cellular phones are made silent and only used for TTX activities when requested, use the microphone to speak, etc.). Table arrangements (if any) for the exercise should try to maximize the interaction between the Facilitator and participants. During the exercise, Facilitators need constantly to be aware of time constraints, notifying participants about progress and moving the discussion toward completion of exercise objectives when time is running short.

**Facilitator Note.** This guide is designed to provide the reader a quick and easy-to-read list of reminders and suggestions on effectively serving as a Facilitator for discussion-based exercises such as TTXs. It is intended to be read well before the day of the exercise. **Much of the Handbook’s content is speaking to Facilitator common norms and best practice, rather than the TTX per se.**

About this Facilitator Handbook

- This Facilitator Handbook is intended to provide Facilitators with the information required to effectively facilitate exercise discussions and ensure effective objective driven play.
- This document contains the information in the Situation Report (SitRep) provided to exercise Players and Observers and has been augmented with additional information intended only for the Facilitator. This supplementary information has been formatted for easy identification as shown in the text box below.
- Under no circumstance should this document or the supplementary information contained within be provided to Players in any form. Failure to comply may disqualify Players from participating in the exercise.

**Facilitator Note:** This manual contains information from the SitRep augmented with supplementary information that can be found in text boxes like this one.

**Table of Contents**

[Exercise Overview 1](#_Toc425847537)

[Exercise Name 1](#_Toc425847538)

[Exercise Dates 1](#_Toc425847539)

[Scope 1](#_Toc425847540)

[Mission Area(s) 1](#_Toc425847541)

[Core Capabilities 1](#_Toc425847542)

[Objectives 1](#_Toc425847543)

[Scenario 2](#_Toc425847545)

[General Information 3](#_Toc425847548)

[Exercise Objectives and Core Capabilities 3](#_Toc425847549)

[Participant Roles and Responsibilities 3](#_Toc425847550)

Facilitating a Tabletop [Exercise 5](#_Toc425847551)

Setting the Stage  [5](#_Toc425847551)

Ways to Involve All Participants [5](#_Toc425847551)

Controlling and Sustaining Action [5](#_Toc425847551)

[Exercise Structure 5](#_Toc425847551)

[Exercise Guidelines 7](#_Toc425847552)

[Exercise Assumptions and Artificialities 7](#_Toc425847553)

[CREID Network Tools and Other Resources 7](#_Toc425847554)

[Non-CREID Network Tools and Other Resources 8](#_Toc425847554)

[Exercise Evaluation 8](#_Toc425847554)

Sessions [A: Outbreak Detected 9](#_Toc425847555)

[Key Issues 9](#_Toc425847559)

[Questions & Prompts 10](#_Toc425847560)

Wrap-up for Session A [11](#_Toc425847560)

Session B[: Outbreak Evolves 12](#_Toc425847561)

[Key Issues 12](#_Toc425847565)

[Questions & Prompts 12](#_Toc425847566)

Wrap-up for Session B [13](#_Toc425847566)

TTX Debrief and Key-Takeaways [14](#_Toc425847567)

Annex A: Exercise Structure & Participants [15](#_Toc425847567)

Exercise Overview

## Exercise Name

## CREID Network TTX: Outbreak Research Response to Pathogen X

## Exercise Dates

September 21–23, 2022

## Scope

This exercise is a discussion-based tabletop exercise (TTX), to be conducted in two 2-hrs sessions at Sheraton Baltimore North Hotel, 903 Dulaney Valley Road, Towson, MD 21204. Exercise play is limited to CREID Network members, i.e., staff associated with CREID Network research centers (RCs) and research sites (RSs), the CREID Coordinating Center, the External Advisory Committee, the NIAID/DMID CREID Program team and invited NIAID observers.

## Mission Area(s)

[Prevention, Protection, Mitigation, Response, and/or Recovery]

## Core Capabilities

Situational Assessment; Network, Infrastructure, Tools, and Resources; ORR Planning and Coordination; Collaboration; Communications and Knowledge Management; Pathogen Research Roadmaps.

## Objectives

***The primary goal of the TTX is to strengthen the CREID Network’s ability to identify and document gaps, weaknesses, strengths, and facilitators for launching timely, effective, and responsive research when an outbreak of any given priority pathogen or pathogen-of-interest occurs***. Specific cross-cutting objectives are:

- Build cross-Network capacity to plan and implement outbreak related research by facilitating knowledge-sharing, relationship-building, and collaboration.
- Increase awareness and test functionality of existing Network tools/resources.
- Test assumptions about the availability and functionality of resources at Research Centers (RCs) and Research Sites (RSs), including MTAs and DUAs.
- Identify, document, and share “unknown” resources, particularly at the RCs and RSs, that can help facilitate outbreak related research.
- Identify and document unmet needs and knowledge gaps, particularly at the RCs and RSs so that responsive solutions can be developed.
- Work collectively to identify research opportunities and develop proactive research agendas that address evidence gaps (i.e., what are the questions that have not yet been answered?).
- Apply findings to prioritize where and how to direct CREID Network resources.

**Facilitator Note:** Remind participants that the TTX objectives were developed during the exercise planning process for very specific reasons and the success of the exercise hinges on focusing discussion on them. As a reminder to you, the TTX objectives are what drive exercise play. Discussion should be redirected to address the objectives listed above as required throughout the TTX.

## Scenario

The CREID Network TTX consists of a fictional infectious disease outbreak of a pathogen X in several countries that the CREID Network is active in, with epidemiological characteristics of recent outbreaks reported globally in the last few years.

Large clusters of cases are reported in DRC, Guinea, Sierra Leone and Uganda; the majority of cases have a history of recent animal exposure; there seems to be some epidemiological linkages among cases from different countries. Clinical symptoms range from fever and malaise to rash, swollen lymph nodes, cough, anorexia, myalgia, nausea, and skin lesions; it is unclear whether cases have co-infections and immunocompetent.

General Information

## Exercise Objectives and Core Capabilities

The exercise objectives below describe the intent of the exercise. The objectives are linked to core capabilities, which are distinct critical elements necessary to achieve the specific mission area(s). The objectives and aligned core capabilities were selected by the Exercise Planning Team.

**Exercise Objective:** Build cross-Network capacity to plan and implement outbreak related research by facilitating knowledge-sharing, relationship-building, and collaboration *[Core Capability:* *Situational Assessment; ORR Planning and Coordination; Collaboration; Communications and Knowledge Management].*

**Exercise Objective:**  Increase awareness and test functionality of existing Network tools/resources *[Core Capability:* *Network, Infrastructure, Tools, and Resources; Communications and Knowledge Management].*

**Exercise Objective:** Test assumptions about the availability and functionality of resources at Research Centers (RCs) and Research Sites (RSs), including MTAs and DUAs. *[Core Capability: Network, Infrastructure, Tools, and Resources; Collaboration; Communications and Knowledge Management].*

**Exercise Objective:** Identify, document, and share “unknown” resources, particularly at the RCs and RSs, that can help facilitate outbreak related research. *[Core Capability: Situational Assessment; Network, Infrastructure, Tools, and Resources; ORR Planning and Coordination; Collaboration; Communications and Knowledge Management]*.

**Exercise Objective:** Identify and document unmet needs and knowledge gaps, particularly at the RCs and RSs so that responsive solutions can be developed *[Situational Assessment; ORR Planning and Coordination; Collaboration; Pathogen Research Roadmaps].*

**Exercise Objective:** Work collectively to identify research opportunities and develop proactive research agendas that address evidence gaps (i.e., what are the questions that have not yet been answered *[Core Capability: Situational Assessment; ORR Planning and Coordination; Collaboration; Pathogen Research Roadmaps].*

## Participant Roles and Responsibilities

The term *Participant* encompasses many groups of people, not just those playing in the exercise. Groups of participants involved in the exercise, and their respective roles and responsibilities, are as follows:

- **Players.** Players are individuals who have an active role in discussing or performing their regular roles and responsibilities during the exercise. Players discuss or initiate actions in response to the simulated disease outbreak.
- **Facilitators.** Facilitators provide situation updates and moderate discussions. They also provide additional information or resolve questions as required. Key TTX Planning Team members also may assist with facilitation as subject matter experts during the exercise. Facilitators are not expected to have or provide answers to all of the Players’ questions. One of the facilitators in each group should also serve as the timekeeper.
- **Reporters.** Reporters are assigned to observe and document Player discussions, including how and if those discussions conform to CREID Network resources, tools, policies, and procedures.
- **Support Staff.** The TTX Support Staff includes individuals who perform administrative and logistical support tasks during the exercise (e.g., registration).
- **Observers.** Observers do not directly participate in the exercise.

**Facilitating a Tabletop Exercise**

The facilitator has a number of responsibilities, including:

- Introducing the narrative
- Encouraging problem solving
- Controlling the pace and flow of the exercise
- Stimulating discussion and drawing answers and solutions from the group (rather than supplying them)

**Setting the Stage**

The opening remarks and activities influence the whole exercise experience. Participants need to know what to expect, and to feel comfortable being participants. Consider including the following elements at the start of your exercise:

- Begin by sincerely welcoming participants and putting them at ease.
- Brief the participants about what will happen. This should include a clear explanation of the exercise’s purpose and objectives, agenda, ground rules, and procedures.
- Start the exercise by reading the first slides of background information.
- Try breaking the ice and being engaging.

**Ways to Involve All Participants**

It is important that everyone participates and that no one person dominates the discussion. Tips for involving all of the participants are summarized below:

- Give extra encouragement to those who are a little tentative.
- Recognize that junior members might be hesitant to comment in front of senior members.
- Avoid the temptation to jump in with the right solutions when participants are struggling. This can hamper discussion. Instead, try to draw out answers from participants. They will be more likely to participate if they feel people are listening intently and sympathetically.
- Model and encourage the behaviors you want from participants.
- Make eye contact with participants.
- Acknowledge comments in a positive manner.
- Be sure to alternate with remote participants (Zoom)

**Controlling and Sustaining Action**

To maintain a high level of interest and to keep everyone involved, the facilitator needs to control and sustain the action. There are several ways to do this.

- Vary the pace. Give messages at different rates, perhaps even giving two at once to increase pace and interest.
- Maintain a balance between talking about a problem to death and moving along so fast that nothing gets settled. Don’t hesitate to control the exercise tightly.
- Watch for signs of frustration or conflict. Always remember that the TTX is an opportunity to evaluate your plan in a no-fault environment, and gaps should be expected. People may be sensitive or inexperienced. If you see mounting frustration or conflict, stop the exercise. Reach into your experience as a discussion leader to help participants resolve conflicts and feel comfortable.
- Keep it low-key. Avoid a bad experience by keeping in mind the low-key nature of the TTX.
- If you spend all of your time on one big problem, maintain interest among participants, and reach consensus, then the tabletop can be considered a success. Push the participants past superficial solutions. A few carefully chosen, open-ended questions can keep the discussion going to a logical conclusion.
- Remember that not everyone will be equally knowledgeable about the plan that is being evaluated.

## Exercise Structure

This exercise will be a multimedia, facilitated exercise. Players will participate in the following two sessions:

- **Session A: Outbreak Detected**
- **Day 1** 1600 – 1615 hrs: Review of the agenda and description of participant roles, followed by an overview of TTX goals and expectations, as well as norms / rules of engagement; highlight that TTX will conclude with a debriefing and self-evaluation. SitRep #1 shared and presented
- **Day 2** 0830 – 0840 hrs: Orientation on WG-specific objectives and session structure
- **Day 2** 0840 – 1020 hrs: Guided discussion about response to SitRep #1.
- **Day 2** 1020 – 1040 hrs: Summary of key take-aways from Session A; Presentation of SitRep #2.


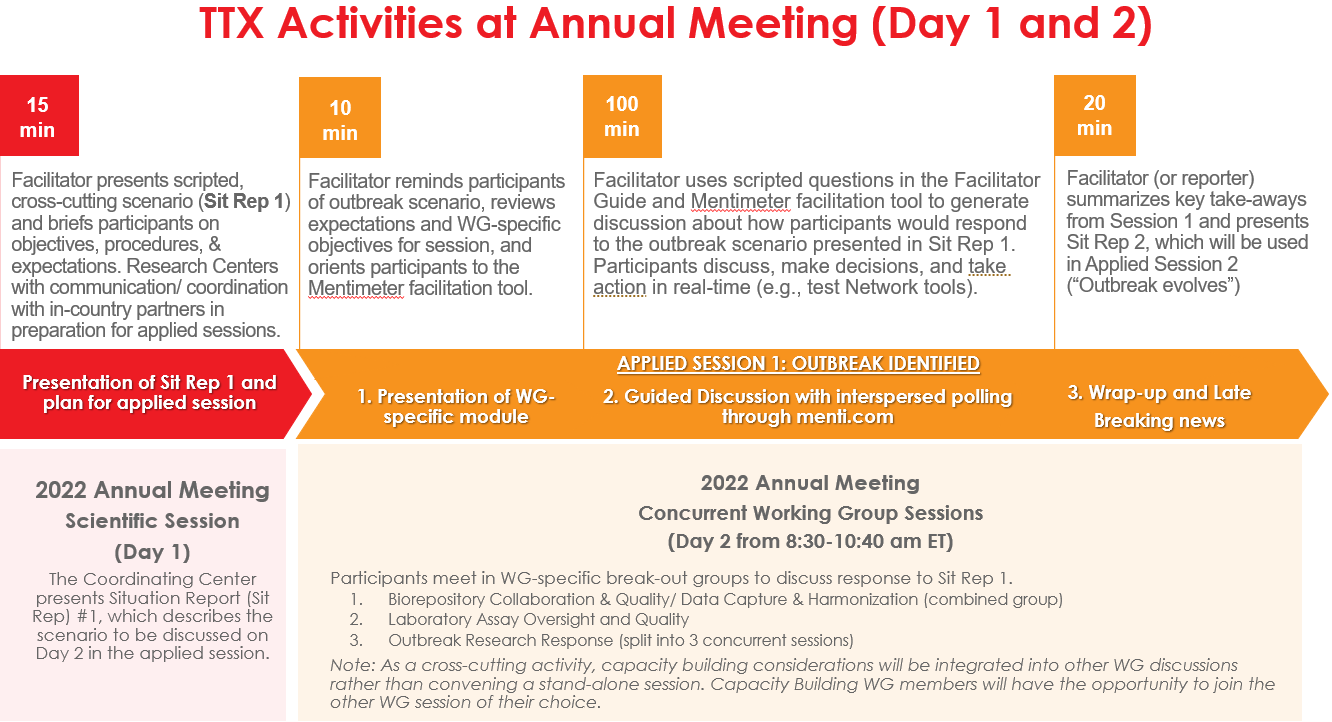


- **Session B: Outbreak Evolves**
- **Day 3** 0830 – 0840 hrs: Review of Session A key take-aways and reminder of SitRep #2
- **Day 3** 0840 – 0930 hrs: Guided discussion about response to SitRep #2.
- **Day 3** 0930 – 0940 hrs: Summary of key take-aways from Session B
- **TTX Debrief and Key Take-Aways**
- **Day 3** 0940 – 1040 hrs: Debriefing (In-situ After-Action Review / Discussion)


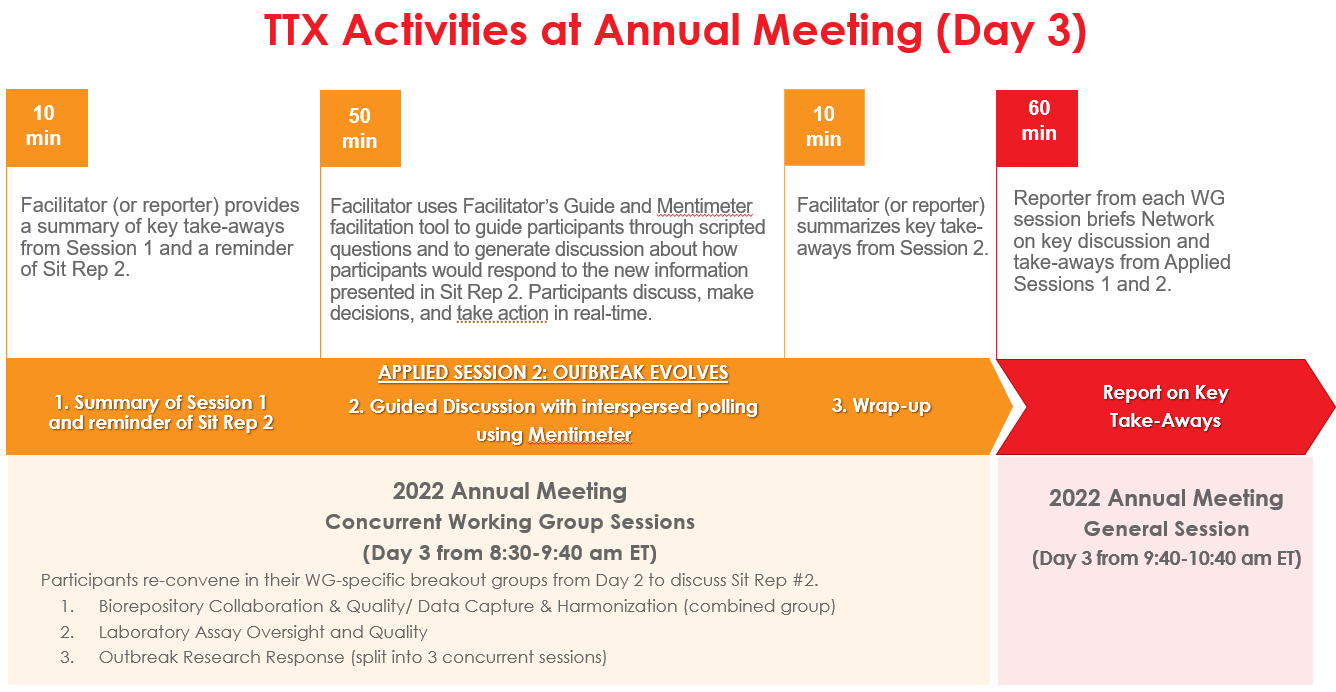


## Exercise Guidelines

- This exercise will be held in an open, low-stress, no-fault environment. Varying viewpoints, even disagreements, are expected.
- Respond to the scenario using your knowledge of current CREID Network capabilities, plans, systems, processes, and tools (i.e., you may use only existing assets), as well as insights derived from your training.
- Decisions are not precedent setting and may not reflect your organization’s final position on a given issue. This exercise is an opportunity to discuss and present multiple options and possible solutions to a simulated disease outbreak.
- Issue identification is not as valuable as suggestions and recommended actions that could improve the timeliness and effectiveness of outbreak research response efforts. Problem-solving efforts should be the focus of this event.

## Exercise Assumptions and Artificialities

In any exercise, assumptions and artificialities may be necessary to complete play in the time allotted and/or account for logistical limitations. TTX participants should accept that assumptions and artificialities are inherent in any exercise and should not allow these considerations to negatively impact their participation. During this TTX, the following apply:

- The exercise is designed to evaluate CREID Network capabilities, plans, systems, processes, and tools rather than individual actions or abilities.
- The exercise scenario is plausible, and events occur as they are presented.
- Participating individuals / organizations may need to balance exercise play with real-world outbreak emergencies; real-world emergencies take priority.

## CREID Network Tools and Other Resources

An objective of the TTX is for participants to familiarize themselves and use the tools and other resources that the CREID Network has developed over the past couple of years. If participants do not mention these in their discussions, facilitators should point to these when guiding participant input and contributions. These tools and other resources include:

- CREID Network Website (external): <https://creid-network.org/>
- CREID Network Website (internal): <https://creid-network.org/secure>
- CREID Network Directory: <https://creid-network.org/secure/directory>
- Network Inventory
- Site Capacity Dashboard: <https://creid-network.org/secure/inventory/site-capacities>, includes tabs for Biorepository, Lab Facility, Outbreak Research, Regulatory, and Shipping & Procurement
- Lab Assay Dashboard: <https://creid-network.org/secure/inventory/lab-assays>
- (Submit) Network Alert and Initial Request Form [here](https://forms.office.com/pages/responsepage.aspx?id=3i78L0RNlEmAgkhzQfpD-wdSghT4YqFHoq7XvY1CgHNUQzZQRFdTOVdESkFQR0dCNEtLQUtRUFFGQi4u).
- (Submit) Network Request Follow-up Form [here](https://forms.office.com/pages/responsepage.aspx?id=3i78L0RNlEmAgkhzQfpD-wdSghT4YqFHoq7XvY1CgHNUNU01Nko4TE9ZNlVCNEc2NVZXS0NONERTSC4u).
- MS Teams Collaborative Space

## Non-CREID Network Tools and Other Resources

An objective of the TTX is for participants to share information from their RCs / RSs and collaborating institutions; additionally, participants should also investigate other publicly available resources to find out more about a certain priority pathogen or pathogen-of-interest. These resources include:

- WHO
- Disease Outbreak News: <https://www.who.int/emergencies/disease-outbreak-news>
- WHO AFRO Outbreaks and Emergencies Bulletin: <https://www.afro.who.int/health-topics/disease-outbreaks/outbreaks-and-other-emergencies-updates>
- ProMed: <https://promedmail.org/>
- HealthMap: <https://www.healthmap.org/en/>
- PubMed: <https://pubmed.ncbi.nlm.nih.gov/>
- ImmPort: <https://www.immport.org/home>
- BEI Resources: <https://www.beiresources.org/>
- NIBSC: <https://www.nibsc.org/>
- CDC:
- Main Page: <https://www.cdc.gov/>
- Permits: <https://www.cdc.gov/cpr/ipp/index.htm>
- Select Agent Program: <https://www.selectagents.gov/sat/list.htm>
- USDA: <https://www.aphis.usda.gov/aphis/resources/permits>

## Exercise Evaluation

Evaluation of the exercise is based on the exercise objectives and aligned capabilities, capability targets, and responses provided by TTX Players. Additionally, Players will be asked to complete feedback in the overall post-Annual Meeting survey. These sources of information, coupled with Facilitator observations and notes, will be used to evaluate the TTX, and compile the After-Action Report (AAR) / Improvement Plan (IP). The ORR team within the CREID Coordinating Center will be responsible for drafting the AAR/IP and circulate to the Facilitators for review and comment within 2 weeks of the completion of the TTX exercise.

Session A: Outbreak Detected

## September 22, 2022: 0830 – 1040 hrs

**Facilitator Note:** Make sure every Player has received and reviewed SitRep #1.

- Scroll to slide in TTX ppt/Menti presentation where SitRep#1 is outlined [we will have paper copies of the SitRep#1 to hand out].

**Facilitator Note:** Point out to participants that the exercise presents an unfolding infectious disease outbreak scenario that is interrupted at several points to allow participants to discuss the situation and to make key decisions.

## Key Issues

- WHO has received reports of a pathogen X-like illness in Brazil, China, Democratic Republic of Congo, France, Guinea, Senegal, Sierra Leone, and Uganda. Clustering of cases has occurred in DRC (n=16), Guinea (n=6), Sierra Leone (n=21), and Uganda (n=7). Among the cases in West and Central Africa, 27 reported a recent history of animal exposure. Isolated cases have also been identified in Brazil, China, France, and Senegal.
- All cases presented with fever and malaise. Other common symptoms include rash, swollen lymph nodes, cough, anorexia, myalgia, nausea, and skin lesions. Clinical reports from the Central Africa cluster (DRC, Uganda) suggest greater disease severity and a higher case fatality rate than other locations. A total of four deaths have been reported (2 in DRC; 1 in Sierra Leone; 1 in Uganda). One death has been linked to an immunocompromised patient. No further information is available about the other deaths.
- Preliminary evidence shows an epidemiological link between the cases in Guinea and Sierra Leone, as well as a separate epidemiological link between cases in DRC and Uganda; there is no known evidence of epidemiological linkages between the cases in West Africa (Guinea, Sierra Leone) and Central Africa (DRC, Uganda).

**Facilitator Note:** The questions below serve as a baseline for discussion. As the exercise Facilitator you are empowered to augment, change, or eliminate them as necessary to drive the discussion towards, or back towards, the stated objectives.

**Facilitator Note:** Make sure to prompt and encourage participants to think of and use CREID Network and non-CREID Network tools and resources when discussing the response to the SitRep.

## Questions & Prompts

We will be using a mix of verbal discussion and online prompts (through Menti platform); questions that could be asked via Menti are in purple.

**Facilitator Note:** the below are illustrative questions and prompts; different CREID working groups, may add specific working group questions and prompts to their Menti template presentations. **Even when using Menti, make sure to prompt Players, as needed (e.g., summarizing Player answers, seeking for clarifications on particular answers, or generally if there are any additional answers).**

- Have Players summarize key events and timeline of pathogen X outbreak described in the SitRep 🡺 *Who wants to summarize key events and timeline of pathogen X outbreak described in the SitRep?*
- Have Players identify critical information gaps based on SitRep 🡺 *What are critical information gaps based on the information provided in the SitRep? What could be potential research questions based on the information provided?*
- Have Players prioritize next steps, whether to get more information to learn from the outbreak or mobilize resources 🡺 *From a CREID Network’s perspective, what do you think should be the next steps to learn more about the outbreak and assess whether CREID should and can engage in ORR?*
- Have Players think of internal RC and RS resources that they have, including the institutional and professional networks that they may beyond CREID *🡺 If you are contacting RSs in affected countries, what type of information would you want to request?*

*This could prompt looking up whether a relevant institutional mapping country brief exists*

- Delegate a group of Players—perhaps from those RCs that have RSs in the DRC, Sierra Leone, and Uganda—create an outbreak notification alert and support request 🡺 *Can a few of you create an outbreak notification alert and support request, one each for DRC, Sierra Leone and Uganda?*
- Stimulate Players to think of resources beyond just their RCs and RSs, but across and even beyond the CREID Network 🡺 *If you think of resources across the CREID Network, what resources would you need and want to mobilize in response to the outbreak described in the SitRep?*

*This could prompt looking up relevant CREID Network directories / inventories / dashboards*

*Would any of the CREID network’s stakeholders be relevant for ORR efforts to respond to the outbreak?*

- Have Players discuss what it would mean to pivot resources to respond to the outbreak, and—if so—how this would be done. 🡺 *How would RCs pivot resources to support research in response to the reported outbreak, and what could possible bottlenecks be?*

Wrap Up for Session A

- How many CREID members outside of your RC have you interacted with in the past 2 hrs?
- Which CREID Network tools or resources did you get exposed to today?
- Which CREID Network tools or resources will you use in the future?
- Summary of Key Take-aways
- How are we feeling?
- Facilitator to present SitRep #2

Session B: Outbreak Evolves

## September 23, 2022: 0830 – 0940 hrs

**Facilitator Note:** Make sure every Player has received and reviewed SitRep #2.

- Scroll to slide in TTX ppt /Menti presentation where SitRep#2 is outlined.

## Key Issues

- A total of 366 cases have been reported (63 suspected; 296 confirmed) in 15 countries globally. This accounts for an additional 7 countries (Cameroon, Liberia, Nigeria, Thailand, Germany, Ecuador, and the US) and an increase of 303 cases since the last report. The majority of cases (58.7%) are found in the Africa region, but large clusters have also emerged in Europe and the Americas.
- More than 50% of cases in Africa report a recent animal exposure. including rodents, bats, monkeys, squirrels, wild pigs, domesticated dogs and cats, and livestock (poultry, pigs, and goats). Outside of Africa, less than 10% of cases report a recent history of animal exposure and only 20% of cases report a recent history of travel.
- 21 deaths have been reported to date, an increase of 17 since the last report. The overall case fatality rate is 5.7%, with a higher rate observed in Central Africa (11.1%).
- Fever is the most common clinical symptom among cases (91.5%), followed by malaise, rash, skin itching, skin lesions, swollen lymph nodes, headache, back pain, sore throat, cough, photophobia, joint stiffness, and difficulty breathing. Based on available data, approximately 35% of cases require hospitalization, although hospitalization rates are much higher in Africa (up to 65%).
- Preliminary laboratory investigation suggests that the unknown illness in Situation Report # 1 is Pathogen X and that there are two separate clades, each with a different pathology.
- Cases appear more prevalent in men than women. Cases range in age from one to 77. In all regions except Africa, the most affected group is 21-30 years old; In the Africa region, the most affected group is 10-21 years old.

**Facilitator Note:** The questions below serve as a baseline for discussion. As the exercise Facilitator you are empowered to augment, change, or eliminate them as necessary to drive the discussion towards, or back towards, the stated objectives.

**Facilitator Note:** Again, as for Session A, make sure to prompt and encourage participants to think of and use CREID Network and non-CREID Network tools and resources when discussing the response to both SitRep.

## Questions & Prompts

We will be using a mix of verbal discussion and online prompts (through Menti platform); questions that could be asked via Menti are in purple.

Based on the information provided, continue the discussion concerning the issues raised in prior day’s discussions with regards to information provided in SitRep #1, and whether decision-making and response changes following release of SitRep #2. Identify any critical issues, decisions, requirements, or questions that should be addressed at this time.

- Have Players summarize how information provided in SitRep#2 clarifies (or not) key events and timeline of pathogen X outbreak described in SitRep#1 🡺 *Who wants to summarize key new information provided in SitRep #2 re events and timeline of pathogen X outbreak?*
- Have Players identify remaining critical information gaps based on both SitReps 🡺 *What are remaining critical information gaps based on the new information provided in SitRep#2? How would you reframe or change the research questions based on the new information provided?*
- Have Players prioritize next steps, whether to get additional information to learn from the outbreak or mobilize resources 🡺 *From a CREID Network’s perspective, are the next steps discussed yesterday still valid in light of the additional information provided in SitRep#2? Does this change the position whether CREID should and can engage in ORR?*
- Stimulate Players to think of resources beyond just their RCs and RSs, but across and even beyond the CREID Network 🡺 *If you think of resources across the CREID Network, what resources would you need and want to mobilize in response to the outbreak described in both SitReps?*

*This could prompt looking up relevant CREID Network directories / inventories / dashboards*

*Would any of the CREID network’s stakeholders be relevant for ORR efforts to respond to the outbreak?*

- Have Players discuss what it would mean to pivot resources to respond to the outbreak, and—if so—how this would be done. 🡺 *How would RCs pivot resources to support research in response to the reported outbreak, and what could possible bottlenecks be?*
- Would your RC/RS participate in a research effort in response to this effort—if *Yes* how; if *No* why not?

Wrap Up for Session B

- How many CREID members outside of your RC have you interacted with in the past 2 hrs?
- Which CREID Network tools or resources did you get exposed to today?
- Which CREID Network tools or resources will you use in the future?
- Summary of Key Take-aways
- How are we feeling?

TTX Debrief and Key Take-aways

## September 23, 2022: 0940 – 1040 hrs

**Facilitator Note:** **Debrief and Take-aways discussion will be done with the entire group of TTX Participants attending the TTX**. Reporters that will have been in the breakout groups will have captured notes of the discussion, which the Facilitators will have to saliently summarize for this session.

**Facilitator Note:** The questions below serve as a baseline for discussion. As the exercise Facilitator you are empowered to augment, change, or eliminate them as necessary to drive the discussion towards, or back towards, the stated objectives.

## Questions & Prompts

We will be using a mix of verbal discussion and online prompts (through Menti platform); questions that could be asked via Menti are in purple.

- Have Players summarize their experience in participating in the TTX, including highlighting aspects they liked or did not like, aspects that were unclear, and aspects that could be improved on 🡺 *Who wants to share their experience over the past two days, aspects of the exercise that they liked or did not like; aspects that were unclear, and aspects that could be improved on.*
- Ask Players whether the TTX goal has been achieved 🡺 *The primary goal of the TTX was to strengthen the CREID network’s ability to identify and document gaps, weaknesses, strengths, and facilitators for launching timely, effective, and responsive research when an outbreak of any given priority pathogen or pathogen-of-interest occurs—in your view has this goal been achieved?*
- Ask Players whether cross-cutting objectives have been achieved 🡺 *Specific objectives of the TTX were to foster relationship building and engagement across the Network, have CREID Network members familiarize themselves with Network tools and resources,* *and develop potential research ideas—in your view have these objectives been achieved?*

Annex A: Exercise Structure & Participants*

| **Working Group** | **Facilitators** | **Reporters** | **Location** | **Players** | | | | **NIH/NIAID** **Observers** |
| --- | --- | --- | --- | --- | --- | --- | --- | --- |
|  |  |  |  | **Total** | **In-person** | **Virtual** | **DK** |  |
| Biorepository Collaboration and Quality + Data Capture and Harmonization | Tony Moody  Nikos Vasilakis  Nathan Vandergrift  Cecelia Sanchez | Hilary Bouton-Verville | Fitzgerald A South | 83  (27 BR; 46 DH) | 28  (15 BR; 13 DH) | 41  (10 BR; 31 DH) | 4  (2 BR; 2 DH) | 12  (3 in-person/ 8 virtual/ 2 DK) |
| Lab Assays | Greg Sempowski  Bob Garry | Nefer Batsuli, Eric Early | Fitzgerald A North | 55 | 23 | 33 | 2 | 8  (1 in-person/6 virtual/1 DK) |
| Outbreak Research Response (Group 1) | Peter Rabinowitz  Jay Hemingway-Foday | Megan Averill | Warfields | 40 | 40 | 0 | - | 7 |
| Outbreak Research Response (Group 2) | Richard Reithinger  Christine Johnson  Kathy Hanley | Hongying Li | Fitzgerald B/C | 45 | 45 | 0 | - | 8 |
| Outbreak Research Response (Group 3) | Rob Breiman  Souleymane Mboup | Danielle Wagner, Aaron Macoubray | Grason | 87 | 0 | 87 | - | 27 |

***Reflects known participant counts as of 15 September 2022. Actual numbers may change slightly before TTX exercise begins.**
