# Supplementary figures and images for: Use of a pathogen X tabletop exercise to assess the operational response preparedness of an emerging infectious diseases research network
Source: Front Public Health. 2025 Mar 27;13:1551996. doi: 10.3389/fpubh.2025.1551996 (PMC11983644; doi:10.3389/fpubh.2025.1551996)

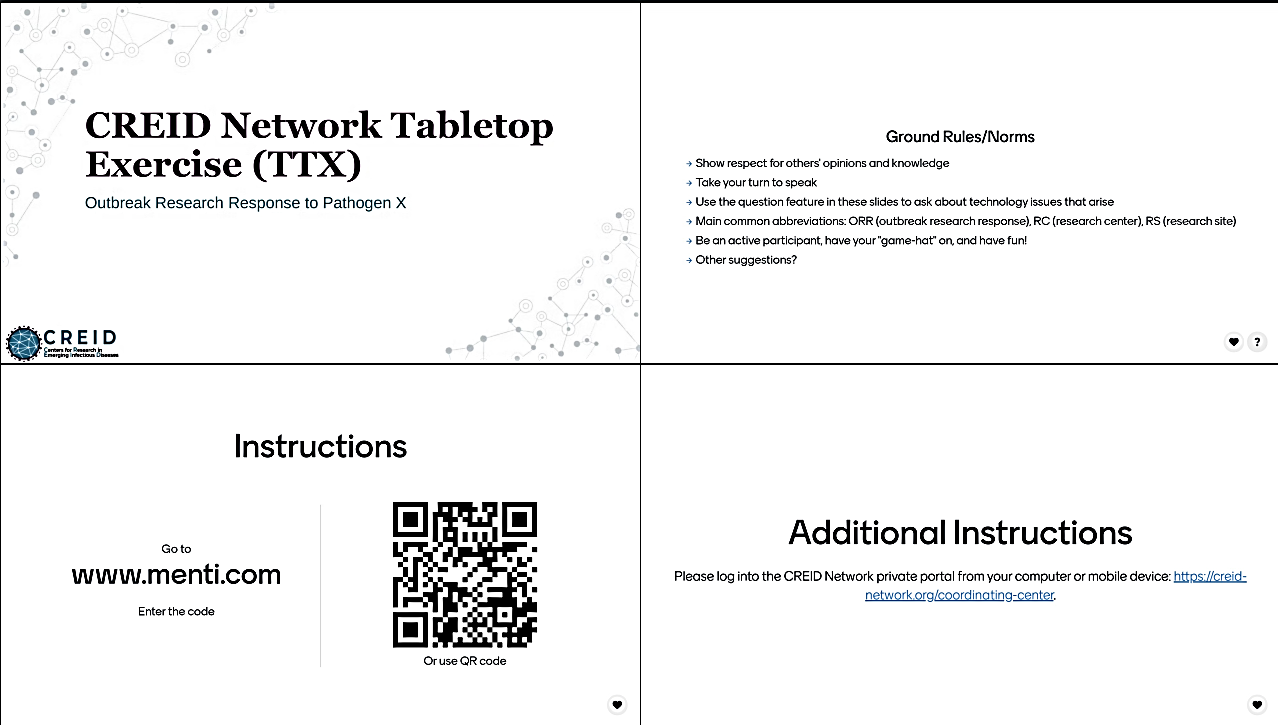

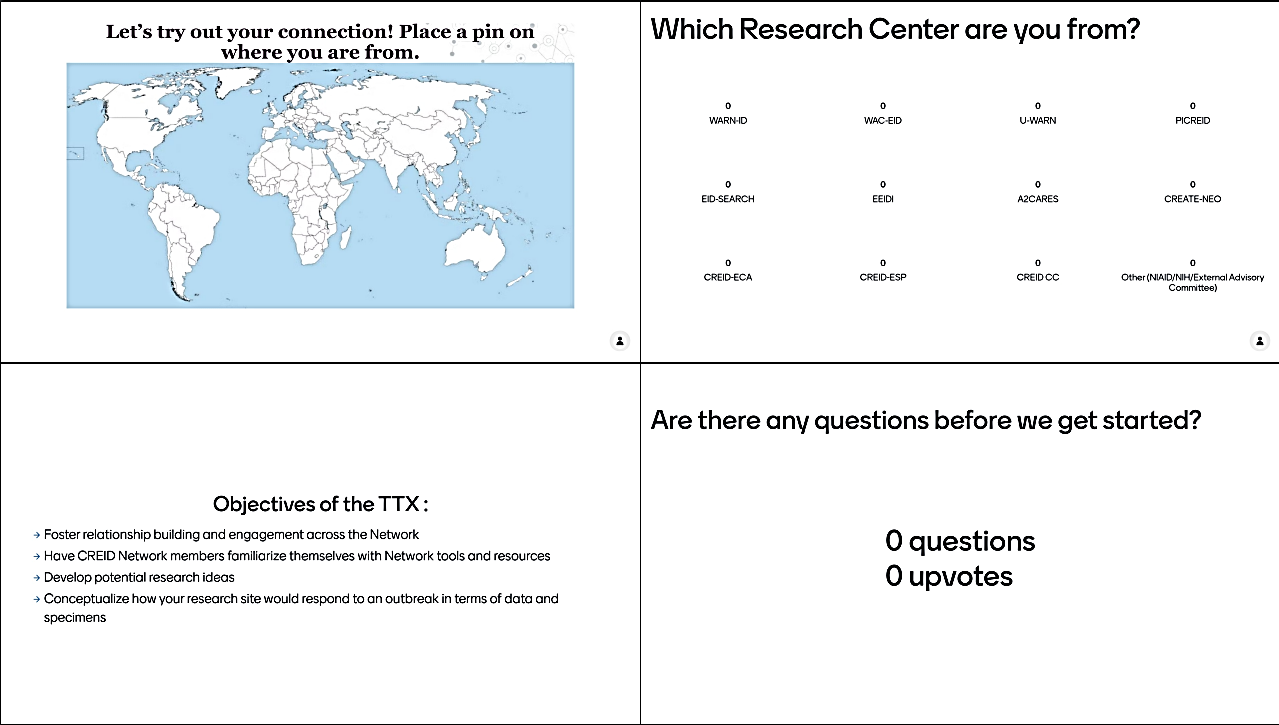

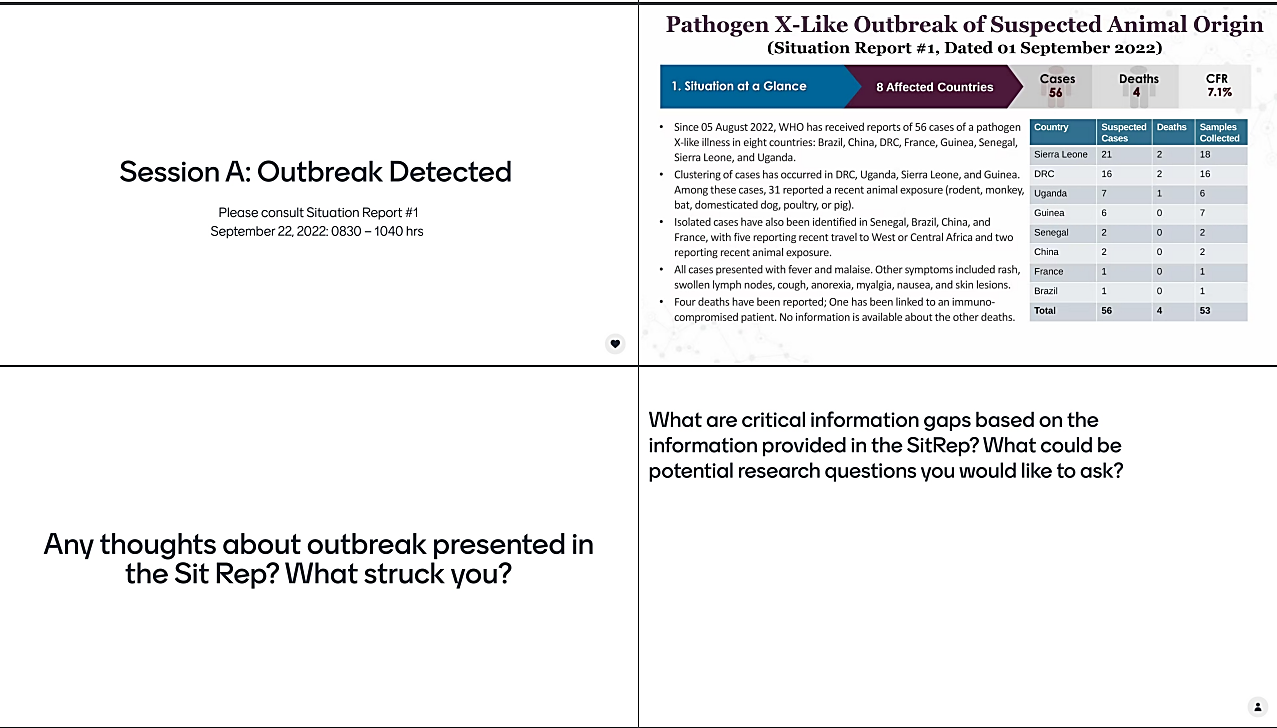

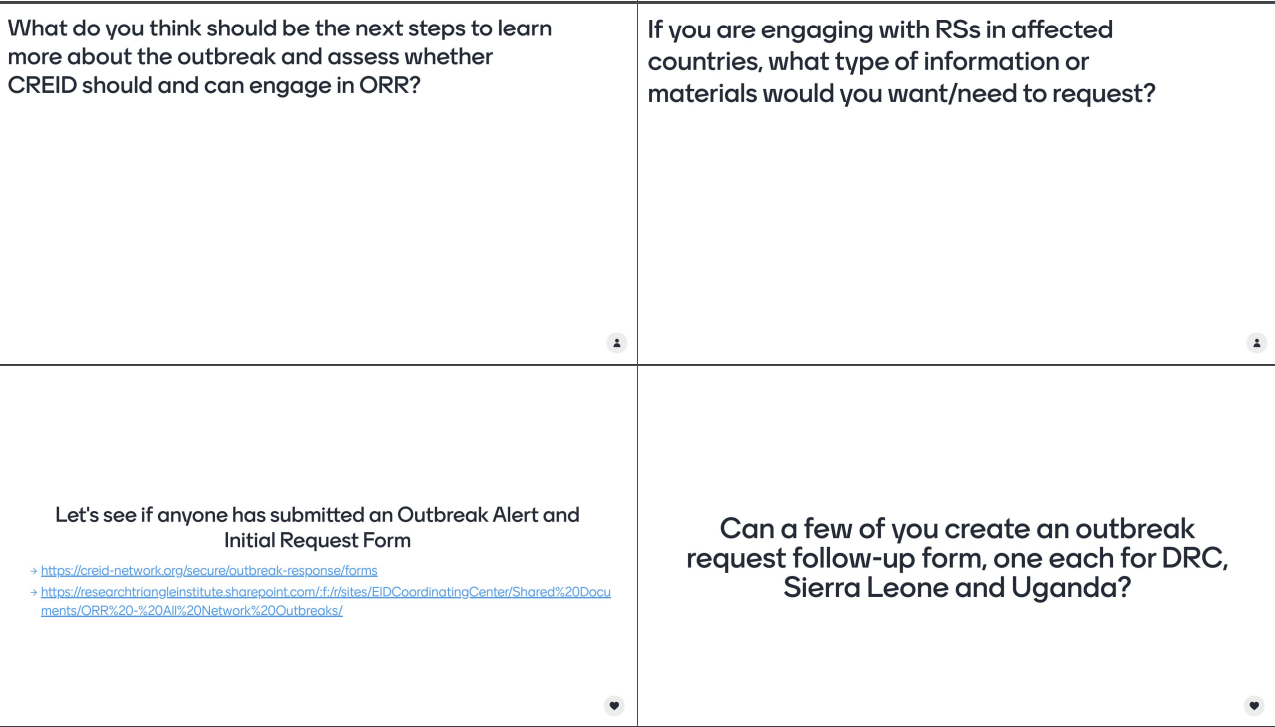

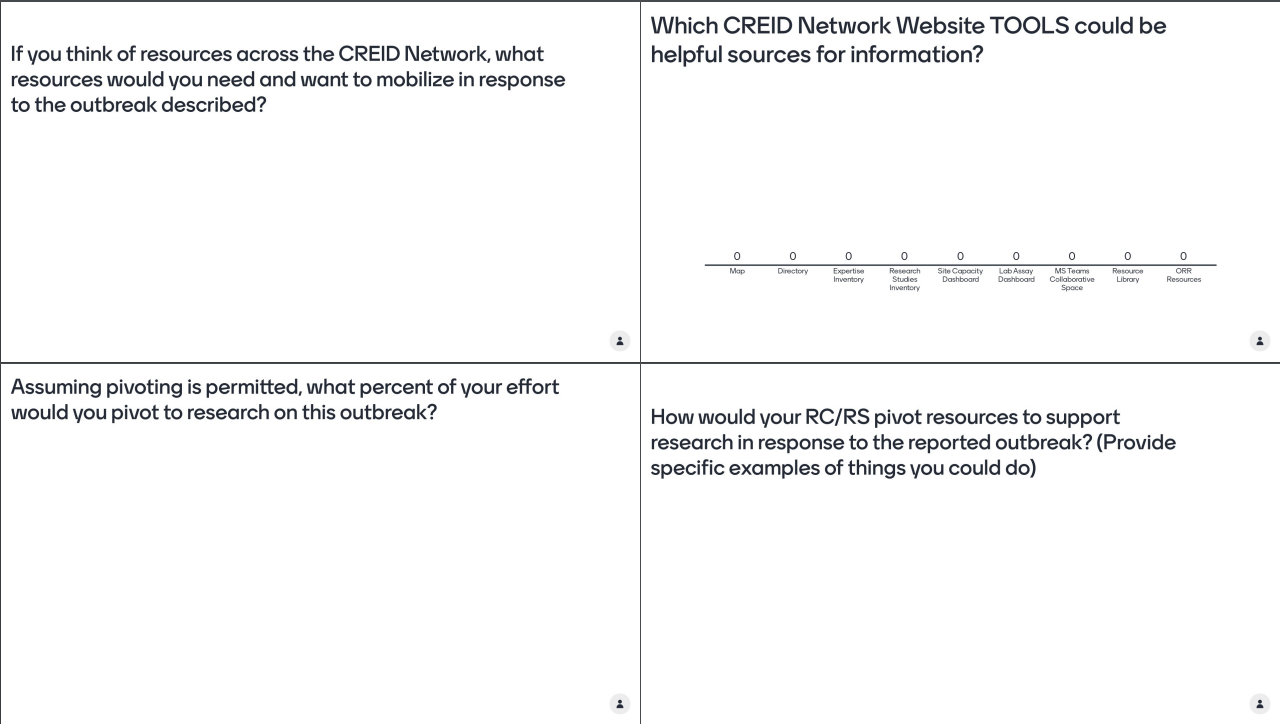

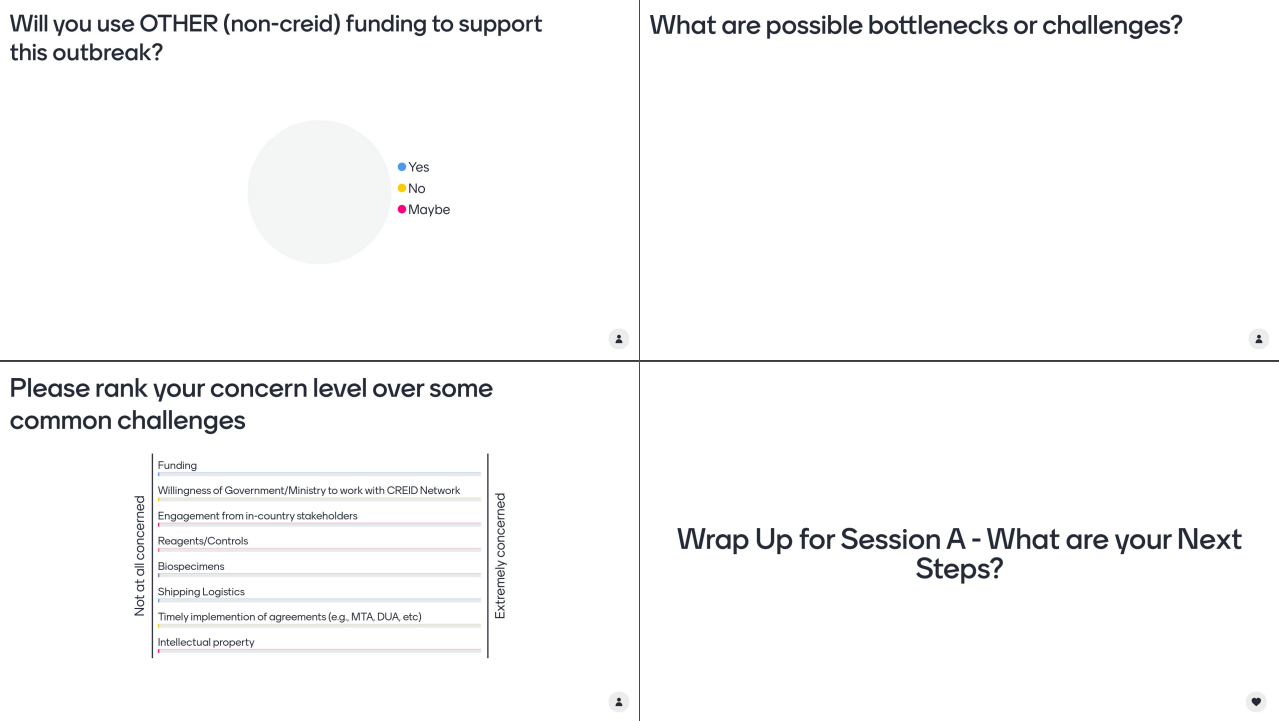

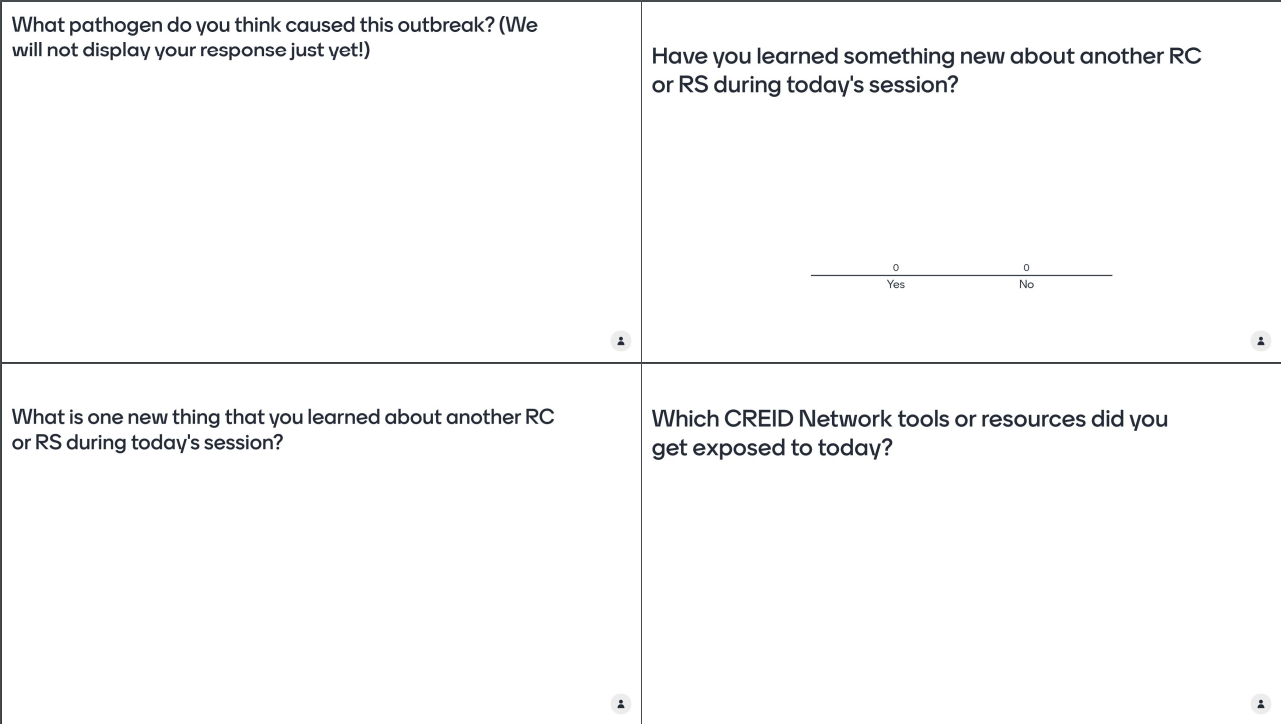

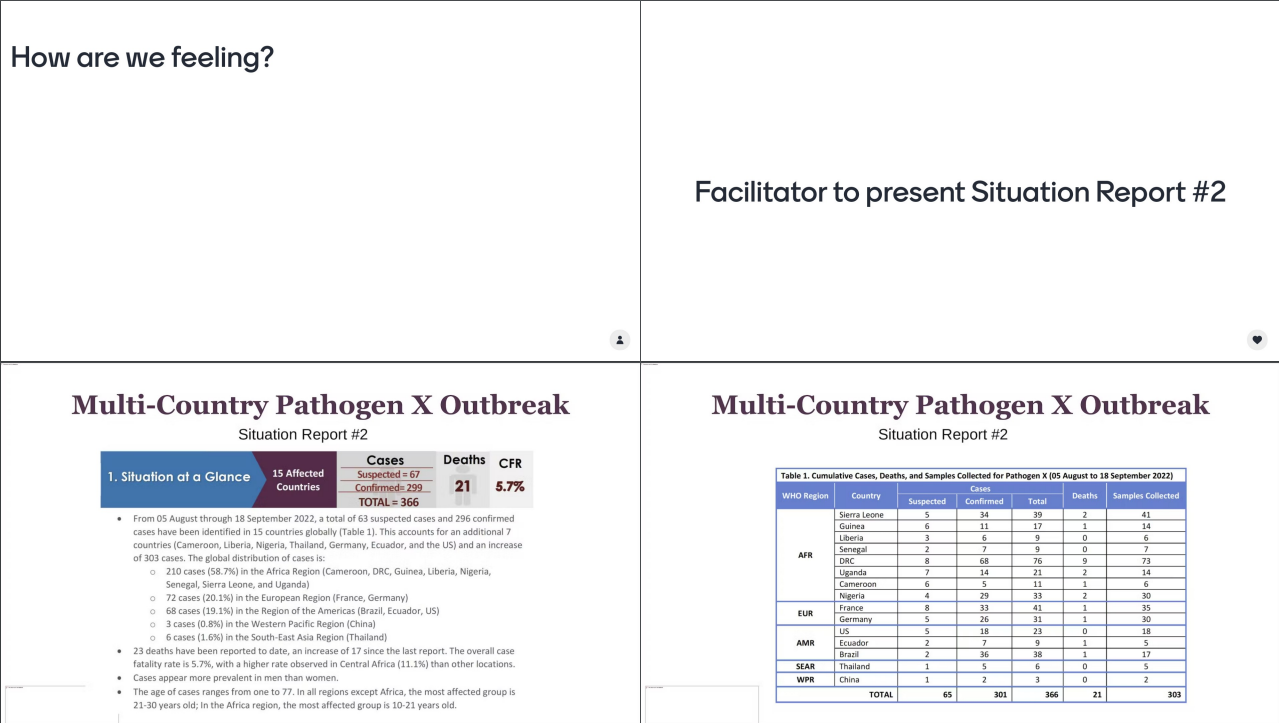

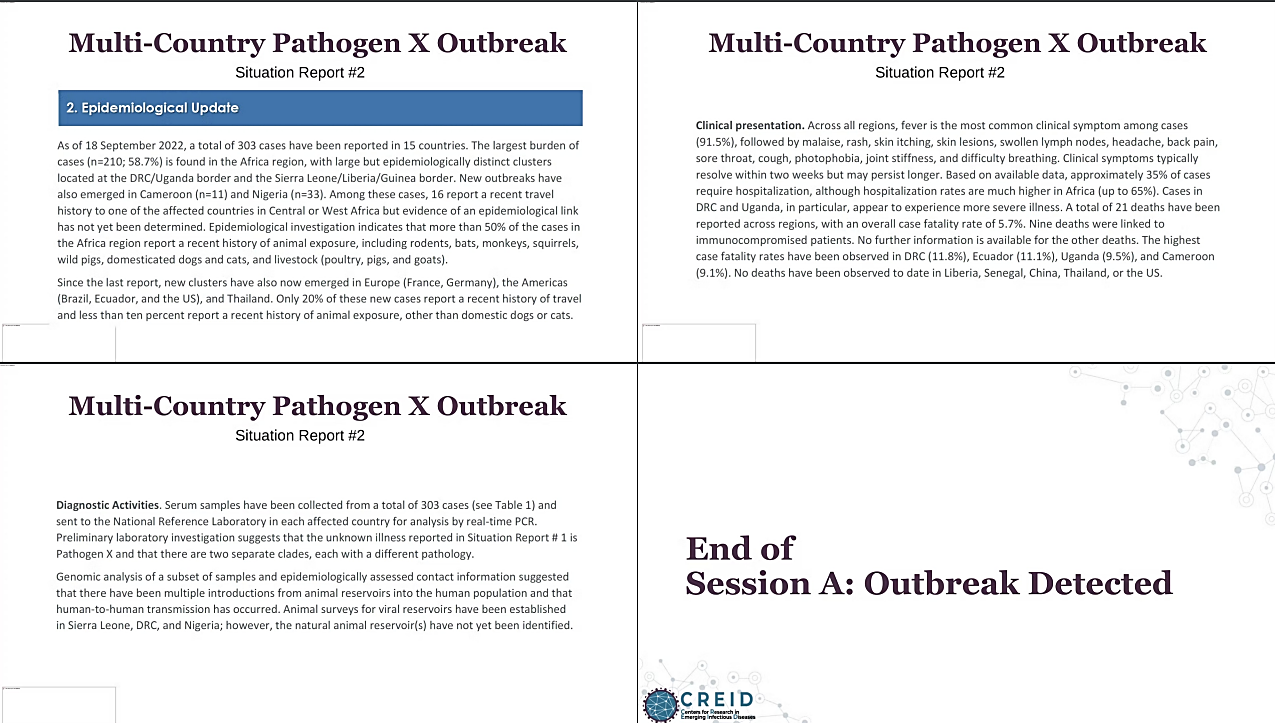

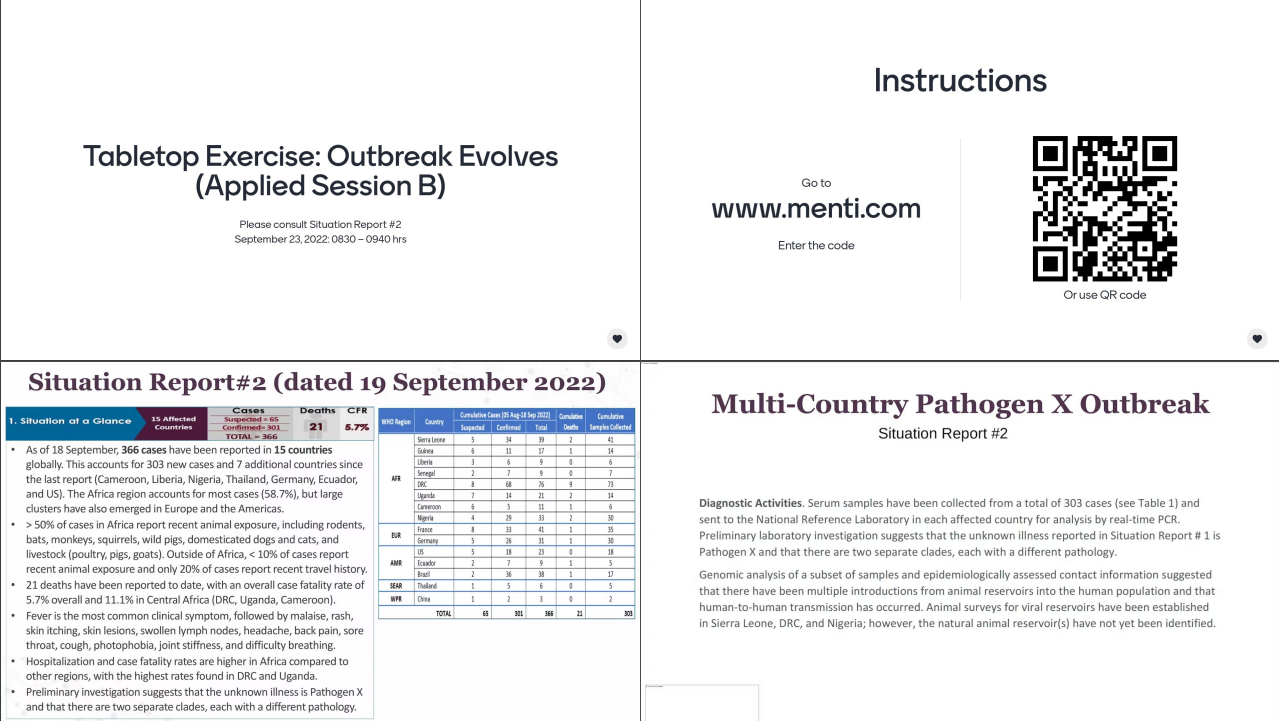

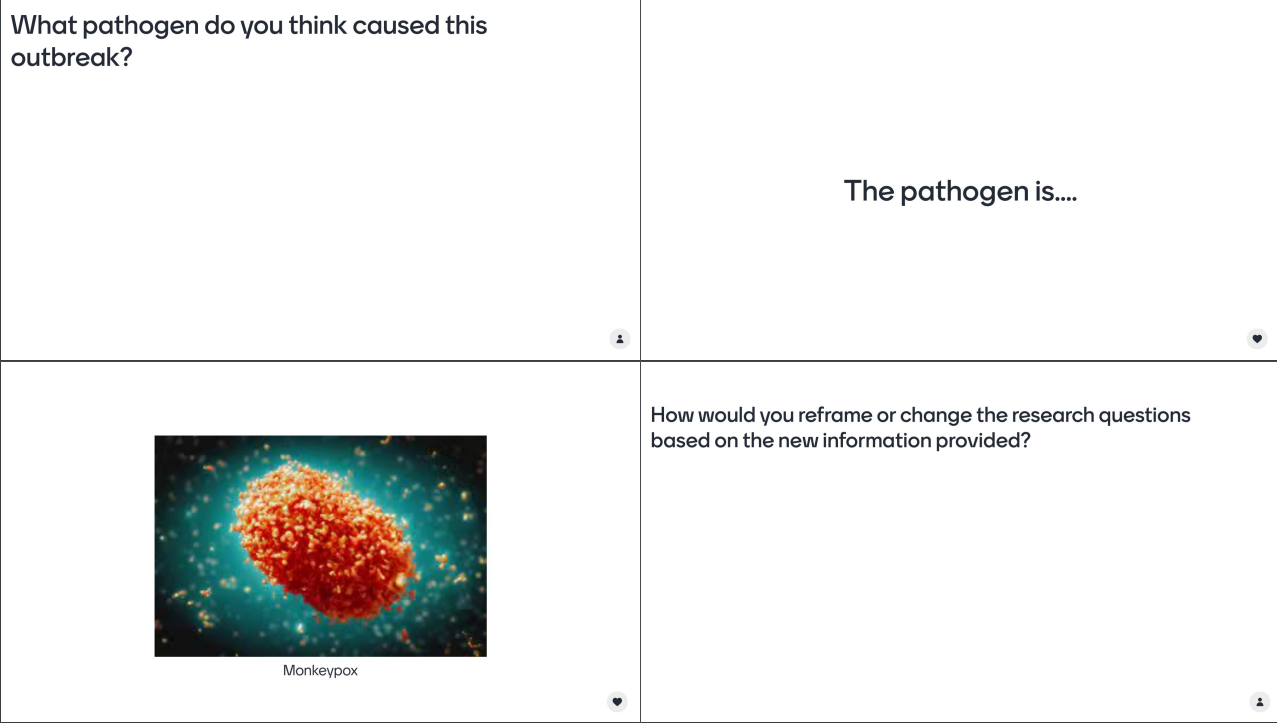

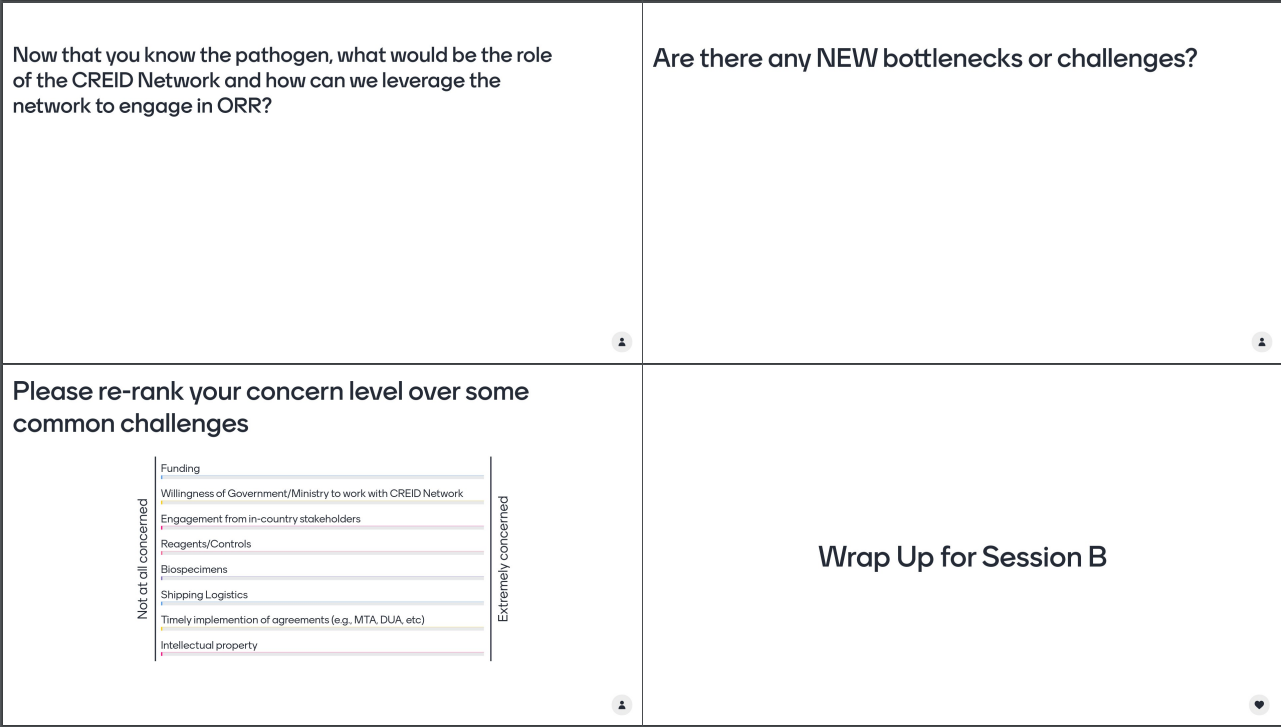

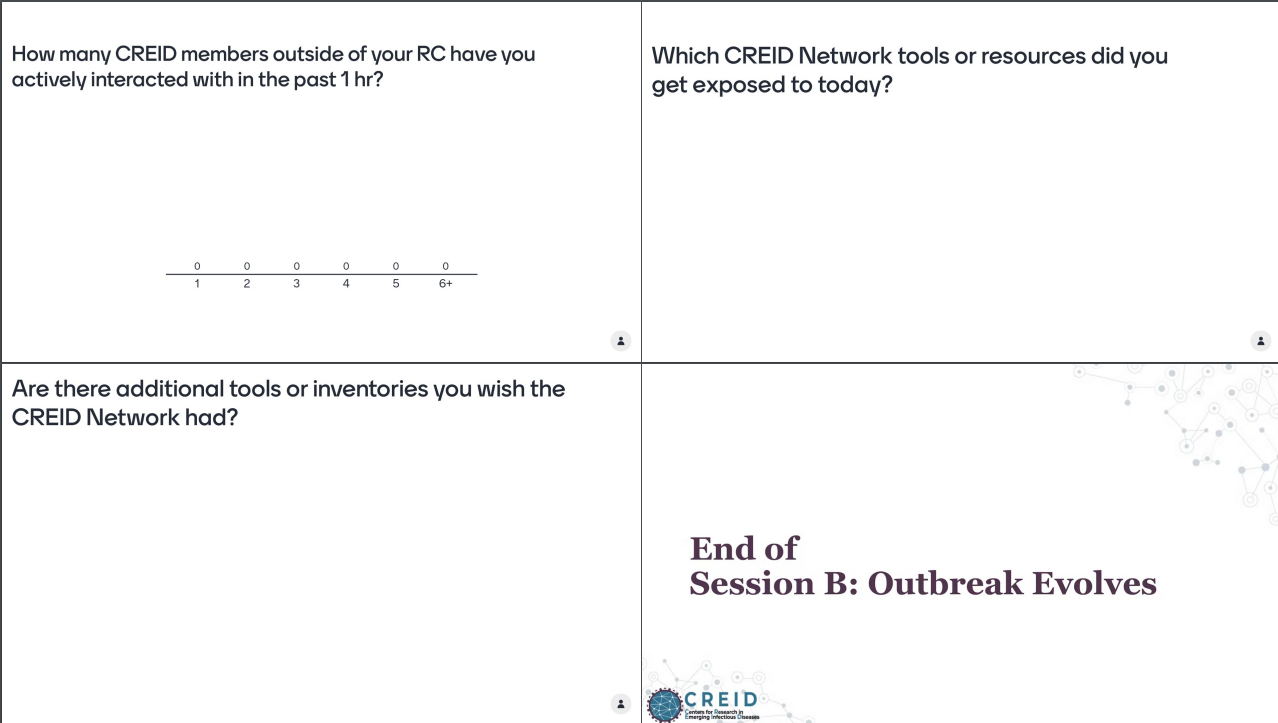

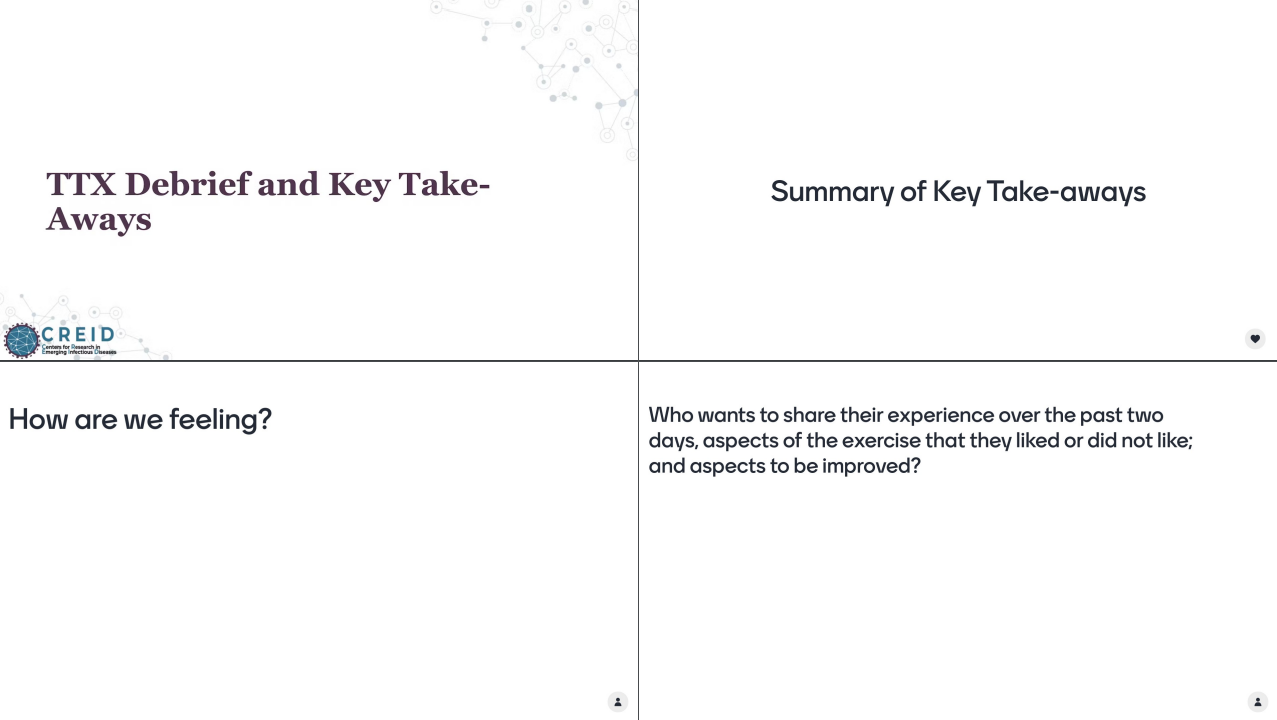

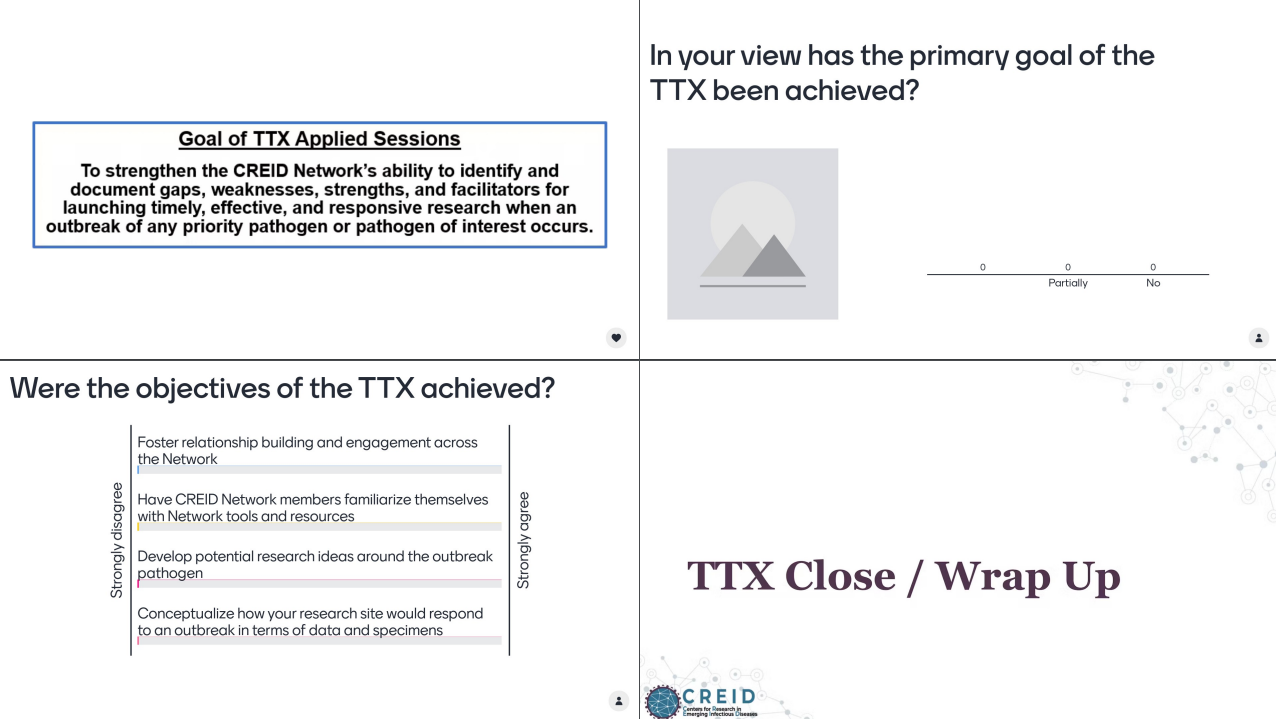

Supplement: Supplementary file 2 [file Data_Sheet_2.docx]
