# Supplementary material for: Use of a pathogen X tabletop exercise to assess the operational response preparedness of an emerging infectious diseases research network
Source: Front Public Health. 2025 Mar 27;13:1551996. doi: 10.3389/fpubh.2025.1551996 (PMC11983644; doi:10.3389/fpubh.2025.1551996)
Supplement: Supplementary file 3 [file Data_Sheet_3.docx]

# **Reporting Template for Applied Session A**

**participant list**

Record # of players by type (as best as possible) using table below

|  | **In-person** | **Virtual** |  |  | **In-person** | **Virtual** |
| --- | --- | --- | --- | --- | --- | --- |
| A2CARES |  |  |  | WAC-EID |  |  |
| CREATE-NEO |  |  |  | WARN-ID |  |  |
| CREID-ECA |  |  |  | CREID CC |  |  |
| CREID-ESP |  |  |  | DMID |  |  |
| EID-SEARCH |  |  |  | Other NIH/NIAID |  |  |
| PICREID |  |  |  | EAC |  |  |

**key discussion points**

**Prompt 1: summarize key events and timeline of pathogen X outbreak described in SitRep #1**

Key Discussion Points

- Add key discussion points here

parking lot (if relevant)

- Add any issues that were sent to the “Parking Lot” by facilitators (i.e., valid points that are raised by participants during the exercise but that risk taking the conversation off topic; these items can be assigned for later discussion to the appropriate persons).

**prompt 2: Identify critical information gaps based on sitrep #1. Potential research approaches**

Key Discussion Points

- Add key discussion points here

parking lot (if relevant)

- Add any issues that were sent to the “Parking Lot” by facilitators (i.e., valid points that are raised by participants during the exercise but that risk taking the conversation off topic; these items can be assigned for later discussion to the appropriate persons).

**Prompt 3: prioritize next steps to learn more about the outbreak and assess CREID engagement**

Key Discussion Points

- Add key discussion points here

parking lot (if relevant)

- Add any issues that were sent to the “Parking Lot” by facilitators (i.e., valid points that are raised by participants during the exercise but that risk taking the conversation off topic; these items can be assigned for later discussion to the appropriate persons).

**prompt 4: Identify rc and rs resources, including institutional and professional networks**

Key Discussion Points

- Add key discussion points here

parking lot (if relevant)

- Add any issues that were sent to the “Parking Lot” by facilitators (i.e., valid points that are raised by participants during the exercise but that risk taking the conversation off topic; these items can be assigned for later discussion to the appropriate persons).

**prompt 5: Create an outbreak notification alert and support request**

Key Discussion Points

- Add key discussion points here

parking lot (if relevant)

- Add any issues that were sent to the “Parking Lot” by facilitators (i.e., valid points that are raised by participants during the exercise but that risk taking the conversation off topic; these items can be assigned for later discussion to the appropriate persons).

**Prompt 6: identify resources to be mobilized in response to outbreak described in sitrep #1**

Key Discussion Points

- Add key discussion points here

parking lot (if relevant)

- Add any issues that were sent to the “Parking Lot” by facilitators (i.e., valid points that are raised by participants during the exercise but that risk taking the conversation off topic; these items can be assigned for later discussion to the appropriate persons).

**prompt 7: discuss how to pivot resources to respond to this outbreak**

Key Discussion Points

- Add key discussion points here

parking lot (if relevant)

- Add any issues that were sent to the “Parking Lot” by facilitators (i.e., valid points that are raised by participants during the exercise but that risk taking the conversation off topic; these items can be assigned for later discussion to the appropriate persons).

**ad-hoc prompt (if needed)**

Key Discussion Points

- Add key discussion points here

parking lot (if relevant)

- Add any issues that were sent to the “Parking Lot” by facilitators (i.e., valid points that are raised by participants during the exercise but that risk taking the conversation off topic; these items can be assigned for later discussion to the appropriate persons).

**ad-hoc prompt (if needed)**

Key Discussion Points

- Add key discussion points here

parking lot (if relevant)

- Add any issues that were sent to the “Parking Lot” by facilitators (i.e., valid points that are raised by participants during the exercise but that risk taking the conversation off topic; these items can be assigned for later discussion to the appropriate persons).

**Wrap-up/key take-aways**

- Summarize key take-aways here

**action items**

1. Enter identified action items here

# **Reporting Template for Applied Session B**

**participant list**

Record # of players by type (as best as possible) using table below

|  | **In-person** | **Virtual** |  |  | **In-person** | **Virtual** |
| --- | --- | --- | --- | --- | --- | --- |
| A2CARES |  |  |  | WAC-EID |  |  |
| CREATE-NEO |  |  |  | WARN-ID |  |  |
| CREID-ECA |  |  |  | CREID CC |  |  |
| CREID-ESP |  |  |  | DMID |  |  |
| EID-SEARCH |  |  |  | Other NIH/NIAID |  |  |
| PICREID |  |  |  | EAC |  |  |

**key discussion points**

**Prompt 1: summarize key events and timeline of pathogen X outbreak described in SitRep #2**

Key Discussion Points

- Add key discussion points here

parking lot (if relevant)

- Add any issues that were sent to the “Parking Lot” by facilitators (i.e., valid points that are raised by participants during the exercise but that risk taking the conversation off topic; these items can be assigned for later discussion to the appropriate persons).

**prompt 2: Identify remaining critical information gaps based on sitrep #2**

Key Discussion Points

- Add key discussion points here

parking lot (if relevant)

- Add any issues that were sent to the “Parking Lot” by facilitators (i.e., valid points that are raised by participants during the exercise but that risk taking the conversation off topic; these items can be assigned for later discussion to the appropriate persons).

**Prompt 3: discuss whether next steps identified in applied session A are still valid**

Key Discussion Points

- Add key discussion points here

parking lot (if relevant)

- Add any issues that were sent to the “Parking Lot” by facilitators (i.e., valid points that are raised by participants during the exercise but that risk taking the conversation off topic; these items can be assigned for later discussion to the appropriate persons).

**Prompt 4: identify resources to be mobilized in response to outbreak described in sitrep #2**

Key Discussion Points

- Add key discussion points here

parking lot (if relevant)

- Add any issues that were sent to the “Parking Lot” by facilitators (i.e., valid points that are raised by participants during the exercise but that risk taking the conversation off topic; these items can be assigned for later discussion to the appropriate persons).

**prompt 5: discuss how to pivot resources to respond to this outbreak**

Key Discussion Points

- Add key discussion points here

parking lot (if relevant)

- Add any issues that were sent to the “Parking Lot” by facilitators (i.e., valid points that are raised by participants during the exercise but that risk taking the conversation off topic; these items can be assigned for later discussion to the appropriate persons).

**prompt 6: discuss whether rc would participate in this research response effort**

Key Discussion Points

- Add key discussion points here

parking lot (if relevant)

- Add any issues that were sent to the “Parking Lot” by facilitators (i.e., valid points that are raised by participants during the exercise but that risk taking the conversation off topic; these items can be assigned for later discussion to the appropriate persons).

**ad-hoc prompt (if needed)**

Key Discussion Points

- Add key discussion points here

parking lot (if relevant)

- Add any issues that were sent to the “Parking Lot” by facilitators (i.e., valid points that are raised by participants during the exercise but that risk taking the conversation off topic; these items can be assigned for later discussion to the appropriate persons).

**ad-hoc prompt (if needed)**

Key Discussion Points

- Add key discussion points here

parking lot (if relevant)

- Add any issues that were sent to the “Parking Lot” by facilitators (i.e., valid points that are raised by participants during the exercise but that risk taking the conversation off topic; these items can be assigned for later discussion to the appropriate persons).

**Wrap-up/key take-aways**

- Summarize key take-aways here

**action items**

1. Enter identified action items here
