# Supplementary material for: Use of a pathogen X tabletop exercise to assess the operational response preparedness of an emerging infectious diseases research network
Source: Front Public Health. 2025 Mar 27;13:1551996. doi: 10.3389/fpubh.2025.1551996 (PMC11983644; doi:10.3389/fpubh.2025.1551996)
Supplement: Supplementary file 4 [file Data_Sheet_4.docx]

# **Situation Reports #1**

**Pathogen X‐Like Outbreak of Suspected Animal Origin**

**Situation Report 01**

**01 September 2022 (Epi Week 36)**


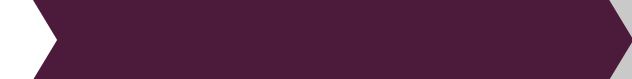

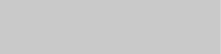

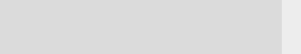

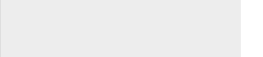

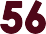

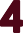

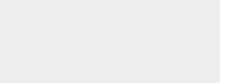

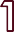

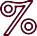


**1. Situation at a Glance**

8 Affected Countries

**CFR**

**Deaths**

**Cases**

- Since 05 August 2022, a total of 56 suspected cases have been identified in 8 countries, with geographic clusters in West Africa (Sierra Leone, Guinea) and Central Africa (DRC, Uganda). Isolated cases have also been identified in Brazil, China, France, and Senegal.


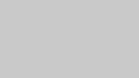

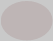

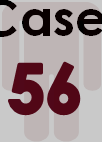

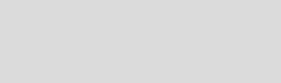

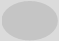

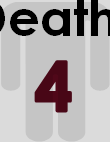


- Four deaths have been reported to date. One death has been linked to an immunocompromised patient. No further information is available about the other deaths.
- Many cases (n=31) report a history of animal exposure, including rodents, bats, monkeys (species unknown), wild pig, domesticated dogs, and livestock (poultry, pigs).
- All cases report fever and malaise. Other common symptoms include rash, swollen lymph nodes, cough, anorexia, myalgia, nausea, and skin lesions.
- Separate and distinct epidemiological linkages have been established among cases in West African cases (Sierra Leone and Guinea) and Central Africa (DRC and Uganda).
- Epidemiological investigation and laboratory analyses are ongoing.


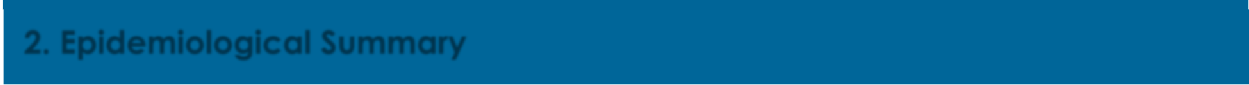


**2. Epidemiological Summary**

Since 05 August 2022, WHO has received reports of a pathogen X‐like illness in Brazil, China, Democratic Republic of Congo (DRC), France, Guinea, Senegal, Sierra Leone, and Uganda (Table 1). Clustering of cases has occurred in West Africa, along the Sierra Leone‐Guinea border, as well as Central Africa, along the DRC‐Uganda border. Among cases reported in West and Central Africa, 29 have a recent history of animal exposure, most commonly rodents. Cases also report exposure through livestock (poultry, pigs), domesticated dogs, or preparation or consumption of bushmeat (bats, monkeys, “bush rats”, wild pigs).

**Table 1. Suspected Cases, Deaths, and Samples for Pathogen X‐like Illness (05 to 31 August 2022)**

| **Country** | **Cases** | **Deaths** | **Samples Collected** |
| --- | --- | --- | --- |
| **Sierra Leone** | 21 | 1 | 18 |
| **DRC** | 16 | 2 | 16 |
| **Guinea** | 6 | 0 | 6 |
| **Uganda** | 7 | 1 | 7 |
| **Senegal** | 2 | 0 | 2 |
| **China** | 2 | 0 | 2 |
| **Brazil** | 1 | 0 | 1 |
| **France** | 1 | 0 | 1 |
| **Total** | 56 | 4 | 53 |

Epidemiological investigation suggests two separate and distinct transmission chains, with the index cases located in Sierra Leone and DRC. The index cases and transmission dynamics are further described in **Section 3 (Description of Cases)**. There is no known evidence of epidemiological linkages between the cases in West Africa and Central Africa.

Isolated cases have also been identified in Brazil, China, France, and Senegal; Among these cases, five have reported recent travel to West or Central Africa and two have reported animal exposure.

**Clinical presentation.** All cases presented with fever and malaise. Other common symptoms include rash, swollen lymph nodes, cough, anorexia, myalgia, nausea, and skin lesions. A total of four deaths have been reported (2 in DRC; 1 in Uganda; 1 in Sierra Leone). One death has been linked to an immunocompromised patient. No further information is available about the other deaths.

**Diagnostic Activities**. Blood samples have been collected from a total of 53 cases (see Table 1) and sent to the National Reference Laboratory in each affected country for diagnosis and viral identification by real‐time PCR. Diagnostic testing results are not yet available. All samples collected in Sierra Leone, Guinea, and Senegal were sent to the Institut Pasteur de Dakar (Dakar, Senegal) for additional diagnostic and confirmatory testing.


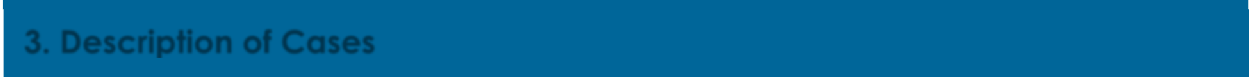


**3. Description of Cases**

***Suspected Clusters in Sierra Leone and Guinea.*** On 05 August 2022, the National IHR Focal Point in Sierra Leone notified WHO of a cluster of cases of a Pathogen X‐like illness in the Kailahun District. All cases presented with fever and malaise; Other symptoms included rash, swollen lymph nodes, skin lesions, and respiratory symptoms. Epidemiological investigation indicates that the index case was a 21‐ year‐old male with a history of animal exposure, including household exposure to rodents and domestic animals or livestock (dogs, poultry, pig) and consumption of bushmeat (bat, “bush rat”). The majority of cases (8) were identified in Yenga, a village located in a remote forest area that sits at the main international border crossing with Guinea. On 15 August 2022, health authorities in Guinea reported a household cluster of three cases presented with symptoms similar to the Sierra Leone cases. The household cluster is located in Nongoa, within the Guéckédou prefecture, and approximately 9 miles from the Sierra Leone border. Epidemiological investigations are ongoing; preliminary information indicates that one of the Guinea cases has an established travel link to affected areas in Sierra Leone and all three Guinea cases have a history of animal exposure, including rodents, bats, monkeys (unknown species), and domesticated animals or livestock. The proximity of the affected areas to international borders, cross‐border movement between the Sierra Leone and Guinea, and the potential transmission of the unknown pathogen between animal vectors and humans poses an increased risk for cross‐border spread. These factors also suggest a high risk at the national and regional level, given that the Kailahun District is well connected to the Guéckédou prefecture in Guinea and Lofa County in Liberia.

***Suspected Clusters in DRC and Uganda.*** On 25 August 2022, the Minister of Health in DRC reported 16 cases of Pathogen X‐like illness in the North Kivu Province. Epidemiological investigation indicates that the suspected index case and majority of suspected cases reside in the Beni Health zone, located on the border with Uganda. This report of the cluster in Beni directly follows the 22 August 2022 announcement by the DRC Minister of Health of a newly identified Ebola virus disease (EVD) outbreak in the same health zone. The suspected index case is a 5‐year‐old male who presented to a public health facility with a 10‐day history of fever, malaise, swollen lymph nodes, and appearance of rashes on his skin. Three household contacts presented with similar symptoms, although the time of symptom onset was less than seven days. Before presenting to the health facility, the child and his family sought care from a traditional health practitioner who provided local herbs for the fever and rash. The suspected index case and his household contacts report a history of animal exposure, including rodents, monkeys of unknown species, and livestock (poultry, goats) within two weeks of symptom onset. The mother of the suspected index case also reports frequent trips to neighboring villages, including several villages in the Kasese District of Uganda to sell bush meat.

***Suspected Cases in Senegal, China, Brazil, and France.*** Isolated cases of Pathogen X‐like disease have also been reported in Senegal, China, Brazil, and France, five cases reported recent travel to West or Central Africa (2 from China and 1 each from Senegal, Brazil, and France). Among these five patients, only those from Senegal and France reported travel to an area with an identified cluster. Two suspected cases (1 from China and 1 from Senegal) also report a recent animal exposure.

# **Situation Report #2**

**Multi‐Country Pathogen X Outbreak**

**Situation Report 02**

**19 September 2022 (Epi Week 39)**

**1. Situation at a Glance**

**21**

**CFR**

**15 Affected Countries**

**Cases**

**Suspected = 65**

**Confirmed= 301**

**TOTAL = 366**

**5.7%**

**Deaths**

**15 Affected Countries**

- From 05 August through 18 September 2022, a total of 366 cases (65 suspected; 301 confirmed) of Pathogen X have been identified in 15 countries globally (Table 1). This accounts for an additional 7 countries (Cameroon, Liberia, Nigeria, Thailand, Germany, Ecuador, and the US) and an increase of 303 cases since the last report. The global distribution of cases is:
  - 210 cases (58.7%) in the Africa Region (Cameroon, DRC, Guinea, Liberia, Nigeria, Senegal, Sierra Leone, and Uganda)
  - 72 cases (20.1%) in the European Region (France, Germany)
  - 68 cases (19.1%) in the Region of the Americas (Brazil, Ecuador, US)
  - 3 cases (0.8%) in the Western Pacific Region (China)
  - 6 cases (1.6%) in the South‐East Asia Region (Thailand)
- 21 deaths have been reported to date, an increase of 17 since the last report. The overall case fatality rate is 5.7%, with a higher rate observed in Central Africa (11.1%) than other locations.
- Cases appear more prevalent in men than women.

| **Table 1. Cumulative Cases, Deaths, and Samples Collected for Pathogen X (05 August to 18 September 2022)** | | | | | | |
| --- | --- | --- | --- | --- | --- | --- |
| **WHO Region** | **Country** | **Suspected** | **Cases**  **Confirmed** | **Total** | **Deaths** | **Samples Collected** |
| **AFR** | Sierra Leone | 5 | 34 | 39 | 2 | 41 |
|  | Guinea | 6 | 11 | 17 | 1 | 14 |
|  | Liberia | 3 | 6 | 9 | 0 | 6 |
|  | Senegal | 2 | 7 | 9 | 0 | 7 |
|  | DRC | 8 | 68 | 76 | 9 | 73 |
|  | Uganda | 7 | 14 | 21 | 2 | 14 |
|  | Cameroon | 6 | 5 | 11 | 1 | 6 |
|  | Nigeria | 4 | 29 | 33 | 2 | 30 |
| **EUR** | France | 8 | 33 | 41 | 1 | 35 |
|  | Germany | 5 | 26 | 31 | 1 | 30 |
| **AMR** | US | 5 | 18 | 23 | 0 | 18 |
|  | Ecuador | 2 | 7 | 9 | 1 | 5 |
|  | Brazil | 2 | 36 | 38 | 1 | 17 |
| **SEAR** | Thailand | 1 | 5 | 6 | 0 | 5 |
| **WPR** | China | 1 | 2 | 3 | 0 | 2 |
| **TOTAL** | | **65** | **301** | **366** | **21** | **303** |

- The age of cases ranges from one to 77. In all regions except Africa, the most affected group is 21‐30 years old; In the Africa region, the most affected group is 10‐21 years old.


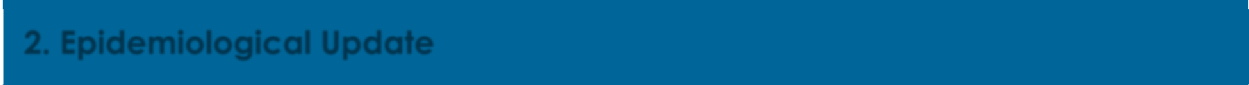


**2. Epidemiological Update**

As of 18 September 2022, a total of 303 cases have been reported in 15 countries. The largest burden of cases (n=210; 58.7%) is found in the Africa region, with large but epidemiologically distinct clusters located at the DRC/Uganda border and the Sierra Leone/Liberia/Guinea border. New outbreaks have also emerged in Cameroon (n=11) and Nigeria (n=33). Among these cases, 16 report a recent travel history to one of the affected countries in Central or West Africa but evidence of an epidemiological link has not yet been determined. Epidemiological investigation indicates that more than 50% of the cases in the Africa region report a recent history of animal exposure, including rodents, bats, monkeys, squirrels, wild pigs, domesticated dogs and cats, and livestock (poultry, pigs, and goats).

Since the last report, new clusters have also now emerged in Europe (France, Germany), the Americas (Brazil, Ecuador, and the US), and Thailand. Only 20% of these new cases report a recent history of travel and less than ten percent report a recent history of animal exposure, other than domestic dogs or cats.

**Clinical presentation.** Across all regions, fever is the most common clinical symptom among cases (91.5%), followed by malaise, rash, skin itching, skin lesions, swollen lymph nodes, headache, back pain, sore throat, cough, photophobia, joint stiffness, and difficulty breathing. Clinical symptoms typically resolve within two weeks but may persist longer. Based on available data, approximately 35% of cases require hospitalization, although hospitalization rates are much higher in Africa (up to 65%). Cases in DRC and Uganda, in particular, appear to experience more severe illness. A total of 21 deaths have been reported across regions, with an overall case fatality rate of 5.7%. Nine deaths were linked to immunocompromised patients. No further information is available for the other deaths. The highest case fatality rates have been observed in DRC (11.8%), Ecuador (11.1%), Uganda (9.5%), and Cameroon (9.1%). No deaths have been observed to date in Liberia, Senegal, China, Thailand, or the US.

**Diagnostic Activities**. Serum samples have been collected from a total of 303 cases (see **Table 1**) and sent to the National Reference Laboratory in each affected country for analysis by real‐time PCR. Preliminary laboratory investigation suggests that the unknown illness reported in Situation Report # 1 is Pathogen X and that there are two separate clades, each with a different pathology.

Genomic analysis of a subset of samples and epidemiologically assessed contact information suggested that there have been multiple introductions from animal reservoirs into the human population and that human‐to‐human transmission has occurred. Animal surveys for viral reservoirs have been established in Sierra Leone, DRC, and Nigeria; however, the natural animal reservoir(s) have not yet been identified.
