# Supplementary material for: Use of a pathogen X tabletop exercise to assess the operational response preparedness of an emerging infectious diseases research network
Source: Front Public Health. 2025 Mar 27;13:1551996. doi: 10.3389/fpubh.2025.1551996 (PMC11983644; doi:10.3389/fpubh.2025.1551996)
Supplement: Supplementary file 5 [file Data_Sheet_5.docx]

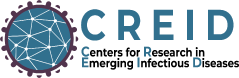


**CREID Network Tabletop Exercise (TTX): Outbreak Research Response to Pathogen X**

After-Action Report

February 28, 2023

on behalf of the CREID Coordinating Center

**Table of Contents**

[**1.** **Activity Summary** 2](#_Toc128397246)

[**2.** **Introduction and Background** 3](#_Toc128397247)

[**3.** **Description of Tabletop Exercise** 4](#_Toc128397248)

[3.1. TTX Goals and Objectives 4](#_Toc128397249)

[3.2. Overall TTX Organization 4](#_Toc128397250)

[3.3. Applied TTX Sessions 5](#_Toc128397251)

[3.4. TTX Scenario 6](#_Toc128397252)

[3.4.1. Scenario A: Outbreak Identified 7](#_Toc128397253)

[3.4.2. Scenario B: Outbreak Evolves 7](#_Toc128397254)

[3.5. Other TTX Tools 7](#_Toc128397255)

[3.6. TTX Attendance 8](#_Toc128397256)

[3.7. TTX Evaluation 8](#_Toc128397257)

[**4.** **Findings: Cross-Network Relationship-Building and Engagement (Objective 1)** 9](#_Toc128397258)

[**5.** **Findings: Familiarity with Network Tools and Resources (Objective 2)** 10](#_Toc128397259)

[**6.** **Findings: Network Readiness for ORR (Objective 3)** 11](#_Toc128397260)

[6.1. Building and Leveraging Partnerships 11](#_Toc128397261)

[6.2. Improving CREID Resources 12](#_Toc128397262)

[**7.** **Findings: Evidence Gaps and Research Priorities for TTX Focus Pathogen (Objective 4)** 14](#_Toc128397263)

[7.1. Other Considerations for ORR 15](#_Toc128397264)

[**8.** **Recommendations** 16](#_Toc128397265)

[8.1. Objective 1: Cross-Network Relationship-Building and Engagement 16](#_Toc128397266)

[8.2. Objective 2: Familiarity with Network Tools and Resources 16](#_Toc128397267)

[8.3. Objective 3: Assess Network Readiness for ORR 17](#_Toc128397268)

[8.4. Objective 4: Evidence Gaps and Research Priorities for TTX Focus Pathogen 18](#_Toc128397269)

[**9.** **Implementation Plan** 19](#_Toc128397270)

# **Activity Summary**

In September 2022, over 150 members of the Centers for Research in Emerging Infectious Diseases (CREID) Network participated in a Tabletop Exercise (TTX) to stress-test the Network’s capacity to prepare for and respond to an emerging pathogen of unknown origin (Pathogen X). The TTX activity is summarized below.

| **Exercise Name** | CREID Network Tabletop Exercise (TTX): Outbreak Research Response to Pathogen X |
| --- | --- |
|  |  |
| **Exercise Dates** | September 21–22, 2022 |
|  |  |
| **Scope** | The TTX simulated an outbreak of an unknown Pathogen X through a series of two applied, 2-hour sessions, which were held in a hybrid format at the CREID Network’s 2022 Annual Meeting. Exercise play included Network members from each of CREID’s 10 Research Centers (RCs), the CREID Coordinating Center (CC), the External Advisory Committee (EAC), and the CREID Program Team from the Division of Microbiology and Infectious Diseases (DMID) at the National Institute of Allergy and Infectious Diseases (NIAID). |
|  |  |
| **Goal** | To strengthen the Network’s ability to identify gaps, weaknesses, strengths, and facilitators for launching timely, well-coordinated, and responsive research when an outbreak of a priority pathogen or pathogen-of-interest occurs. |
|  |  |
| **Objectives** | - Objective 1: Foster cross-Network knowledge-sharing, relationship-building, and collaboration. - Objective 2: Increase familiarity with Network tools and resources. - Objective 3: Assess Network readiness for outbreak-related research. - Objective 4: Identify evidence gaps and research priorities for the TTX’s focus pathogen. |
|  |  |
| **Threat** | Emerging Pathogen X (modeled after mpox; monkeypox) |
|  |  |
| **Scenario** | The TTX was based on two sequentially released, complementary scenarios, structured as Situation Reports, for a fictional outbreak of an unknown Pathogen X of suspected animal origin. Each scenario included an epidemiological summary, case counts, deaths, case fatality rate, clinical presentation, diagnostic activities, and description of cases. The detailed scenarios are found in the **Supplemental Materials.** |
|  |  |
| **Core Capabilities** | The TTX was structured to build the Network’s core capabilities in the following areas:  1. Situational assessment  2. Network infrastructure, tools, and resources  3. Outbreak research response planning, coordination, and collaboration  4. Communications and knowledge management  5. Pathogen research roadmap development |
|  |  |
| **Sponsor** | NIH/NIAID |
|  |  |
| **Participating Organizations** | Over 150 CREID Network members from the CREID CC, DMID, and the 10 CREID RCs, representing diverse geographical regions, areas of expertise and Network roles/responsibilities. See **Supplemental Materials** for full list of represented entities. |
|  |  |
| **Point of Contact** | Jennifer J. Hemingway-Foday ([hemingway@rti.org](mailto:hemingway@rti.org)) and Richard Reithinger (reithinger@rti.org) on behalf of the CREID Coordinating Center (RTI International). |

# **Introduction and Background**

The Centers for Research in Emerging Infectious Diseases (CREID) Network was funded by the National Institute of Allergy and Infectious Diseases (NIAID) in 2020 to enhance global pandemic preparedness and response by building a sustainable, scalable, and adaptable infrastructure for infectious disease research before, during, and after outbreaks. The Network is composed of a central Coordinating Center (CC) and 10 Research Centers (RCs), with approximately 100 research sites (RSs) in more than 30 countries where emerging and reemerging infectious disease (EID) outbreaks are likely to occur. Through the CREID network infrastructure, multidisciplinary teams of investigators collaborate to study potential pandemic pathogens, identify knowledge gaps of host-pathogen interactions, and support critical research efforts to better understand outbreak evolution and perform enabling research to develop critical diagnostics and therapeutics.

The CREID CC provides scalable administrative and management systems, processes, and resources to (1) facilitate CREID Network performance during inter-outbreak periods, and (2) bolster CREID CC research surge response activities during outbreaks. The CREID CC is a partnership between RTI International and the Duke Human Vaccine Institute at Duke University Medical School—leveraging the strengths of each institution to provide network administration, data harmonization, biorepository oversight, and laboratory assays and harmonization to support collaborative global EID outbreak research response (ORR). Additional cross-cutting activities and initiatives include internal and external communication, technology, capacity building, and administration of a Pilot Research Program focused on developing early-stage investigators. A comprehensive online Network Inventory provides real-time data for Network gap analysis to focus and prioritize efforts for collaboration and capacity building.

**Tabletop Exercise (TTX).** To encourage and build a collaborative Network community, share best-practices and resource tools curated by the CC, and to stress test the CREID Network capacity to launch rapid and effective research for future outbreaks, the Network ORR Team designed a TTX that was held during CREID’s 2022 Annual Meeting on September 21–23 in Townson, Maryland. Based on a fictional outbreak simulation, the TTX’s goal was to strengthen the Network’s ability to identify gaps, weaknesses, strengths, and facilitators for launching timely, well-coordinated, and responsive research when an outbreak occurs. The TTX was a Network-wide activity that ran over two, 2-hour hybrid sessions during the meeting. The full CC team, in consultation with RC Principal Investigators (PIs) and ORR Working Group (WG) co-facilitators, developed a common cross-cutting scenario with individualized thematic modules based on priorities identified during the 2021 CREID Annual Meeting and subsequently throughout 2021–2022. The exercise was led and facilitated by the CC’s ORR Team in collaboration with CC/RC WG co-facilitators.

The primary outcome of the effort was to inform a proactive framework for effective, timely, and responsive research when an outbreak occurs. Additional anticipated outcomes that align with the CC and overall Network vision, and 2021 CREID External Advisory Committee (EAC) recommendations included (1) increased Network member communication and development of a collaborative research community, (2) identification of gaps and new research opportunities, (3) removal of barriers to global EID research collaboration, (4) familiarization of Network members with the CC-developed Inventory of capacities and resources, and (5) inclusion of all member voices in a supportive environment.

# **Description of Tabletop Exercise**

## TTX Goals and Objectives

The goals and objectives of the TTX exercise are described in **Exhibit 1**.

**Exhibit 1. Tabletop Exercise Goals and Objectives**


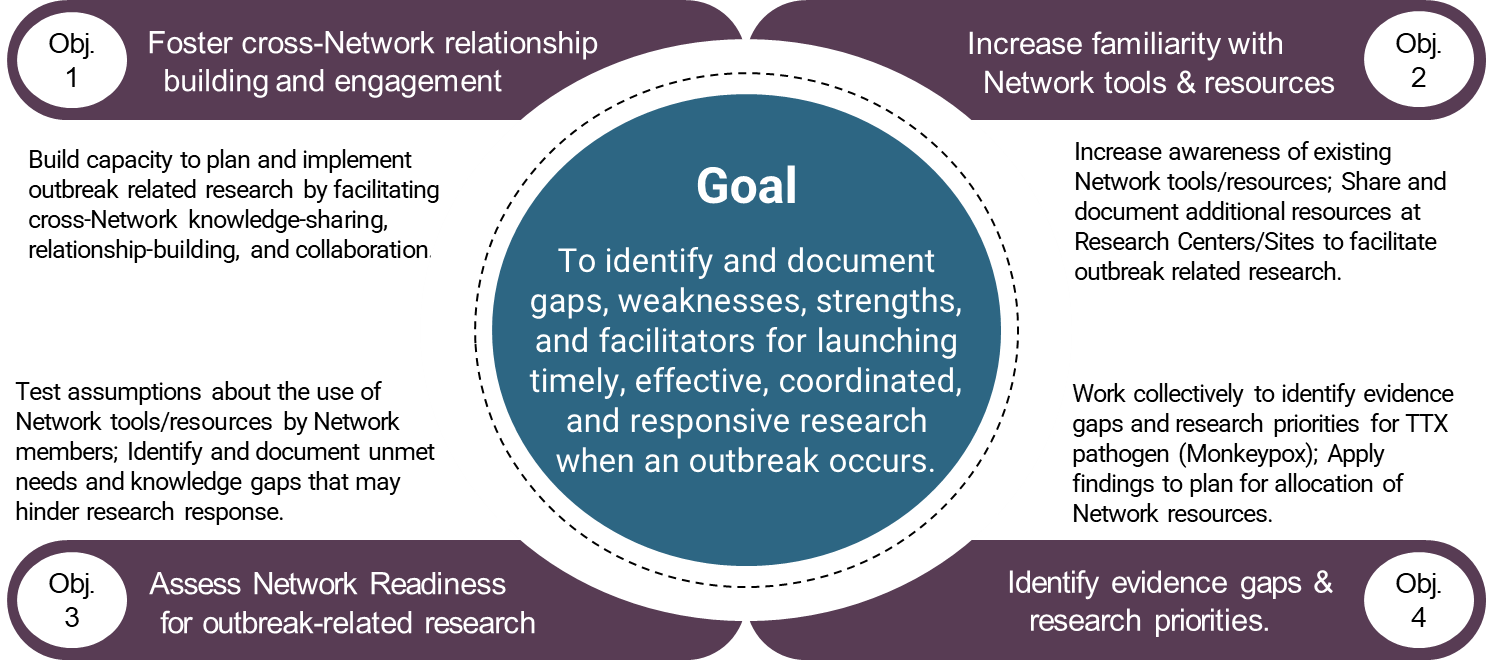


## Overall TTX Organization

The TTX organization consisted of three phases, as outline in **Exhibit 2**: (1) a Strategic Planning Phase (January–August 2022), where a cross-network TTX team was formed, TTX goals and objectives were defined, exercise materials were developed, and CREID Network members were repeatedly sensitized to the exercise during standing WG and Steering Committee meetings prior to the 2022 CREID Annual Meeting; (2) a Deployment Phase (September 2022); and (3) an Analysis and Dissemination Phase of the TTX After Action Report (October 2022-February 2023).

**Exhibit 2. Tabletop Exercise Phases**

| **Phase** | **Key Tasks** |
| --- | --- |
| **I. Strategic Planning**  **(January–August 2022)** | - Form a TTX team, led by the CREID CC’s ORR WG and participation from the CC WG leads for Laboratory Assays, Biorepository, Data Capture and Harmonization, and Capacity Building. - Define TTX goals/objectives, scope, structure, timeline, and evaluation metrics. - Define WG-specific goals and objectives. - Develop messaging to communicate with RCs/RSs about TTX expectations and facilitate engagement. - Develop a cross-cutting scenario in the form of a Situation Report (SitRep) (see **Supplemental Materials**). - Determine the appropriate deployment format and supportive tools/technology. - Determine the composition of Facilitators, Reporters, Players, and Observers for each Working Group breakout group.​ - Develop a Facilitator’s Manual and interactive facilitation tool (see **Supplemental Materials).** - Develop a reporting template to capture feedback during the exercise in a standardized format (see **Supplemental Materials).** |
|  |  |
| **II. Deployment**  **(September 2022)** | - Prepare for deployment:   - Finalize agenda for applied sessions   - Train TTX Facilitators and Reporters to effectively carry out their roles and responsibilities and deploy facilitation and reporting tools   - Plan for logistical needs (e.g., room setup, audio-visual support) - Present Network-wide outbreak scenario at Annual Meeting general session. - Implement applied sessions in five concurrently run breakout groups. - Conduct hotwash at the end of applied sessions to rapidly document key takeaways and action items. - Share hotwash report from each breakout group at general session. |
|  |  |
| **III. Analysis and Dissemination**  **October 2022 – February 2023)** | - Compile and analyze data from applied session notes, hotwash reports, and evaluation form. - Draft report and circulate for review and comments. - Finalize report. - Disseminate report via Network communications channels and Private Portal (Resources Library). |

## Applied TTX Sessions

The TTX was deployed at the CREID 2022 Annual Meeting as two applied, 2-hour hybrid format sessions, each focused on a distinct outbreak scenario, described in two, sequentially released situation reports (SitReps) (**Table 1**; see **Section 3.4.** and **Supplemental Materials**): (1) the first SitRep mimicked a typical outbreak report, where the outbreak was identified, and clinical, epidemiological, and other information shared on the outbreak was uneven/incomplete; and (2) the second SitRep provided more complete and structured clinical, epidemiological, and other information, including answers to questions that arose from the first SitRep.

**Table 1. TTX Agenda**

| **Day** | **Topic** | | **Time** | |
| --- | --- | --- | --- | --- |
| **Day 1** | Presentation of TTX Scenario (SitRep #1) and Overview of Goals and Expectations for Applied Sessions A & B | | 15 minutes | |
| **Day 2** | | **TTX Applied Session A: Outbreak Identified** | | 2 hours |
|  |  | - Presentation of WG-specific guidance | |  |
|  |  | - Guided discussion | |  |
|  |  | - Wrap-up and presentation of SitRep #2 | |  |
| **Day 3** | **TTX Applied Session B: Outbreak Evolves** | | 1.5 hours | |
|  | - Presentation of WG-specific guidance | |  |  |
|  | - Guided discussion | |  |  |
|  | - Wrap-up and summary of key takeaways | |  |  |
|  | Tabletop Exercise: Report out to general session | | 30 minutes | |

A more comprehensive description of the two TTX applied, 2-hour sessions is shown in **Exhibit 3.**

| **Exhibit 3. TTX Activities at Annual Meeting (Days 1 and 2)** |
| --- |
| 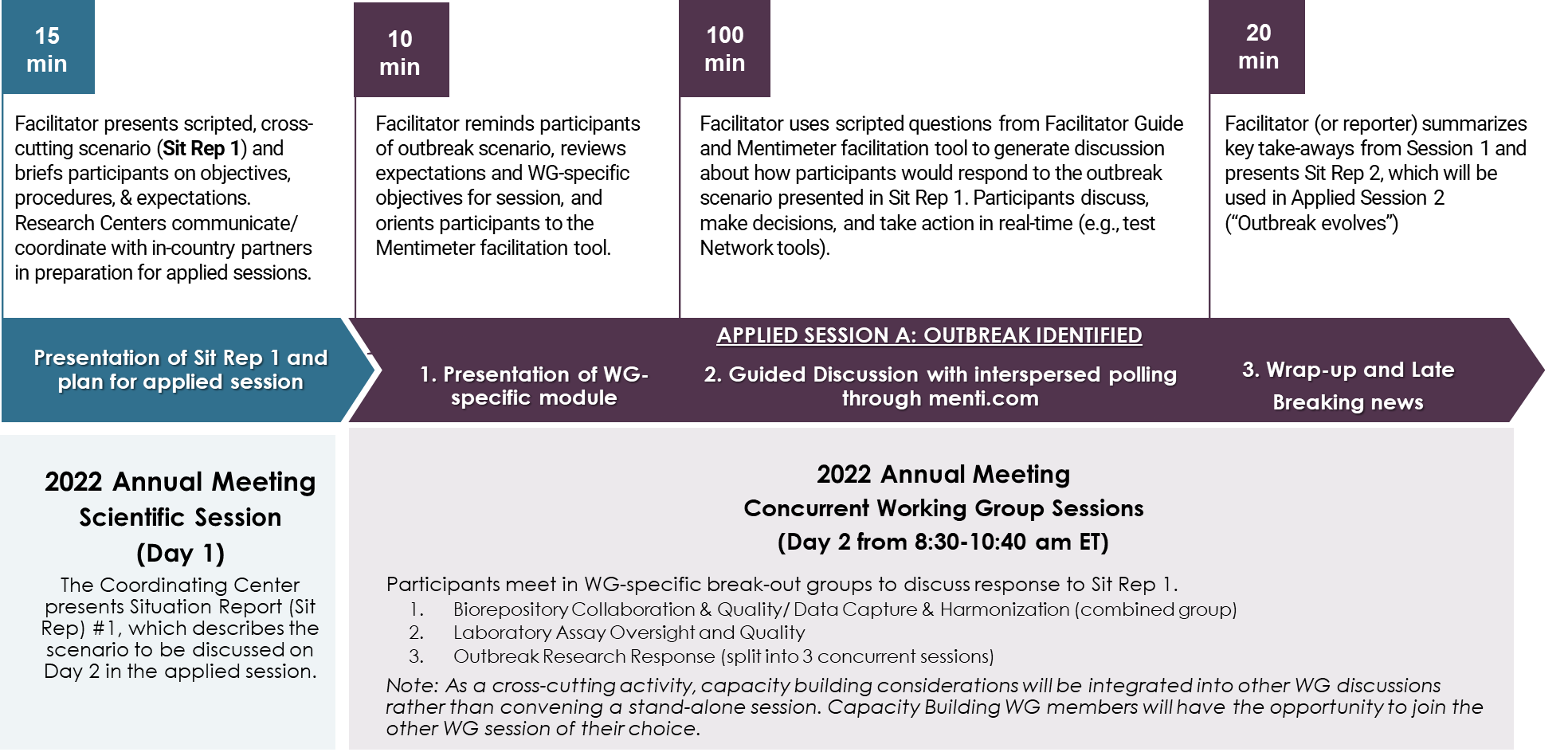 |
| 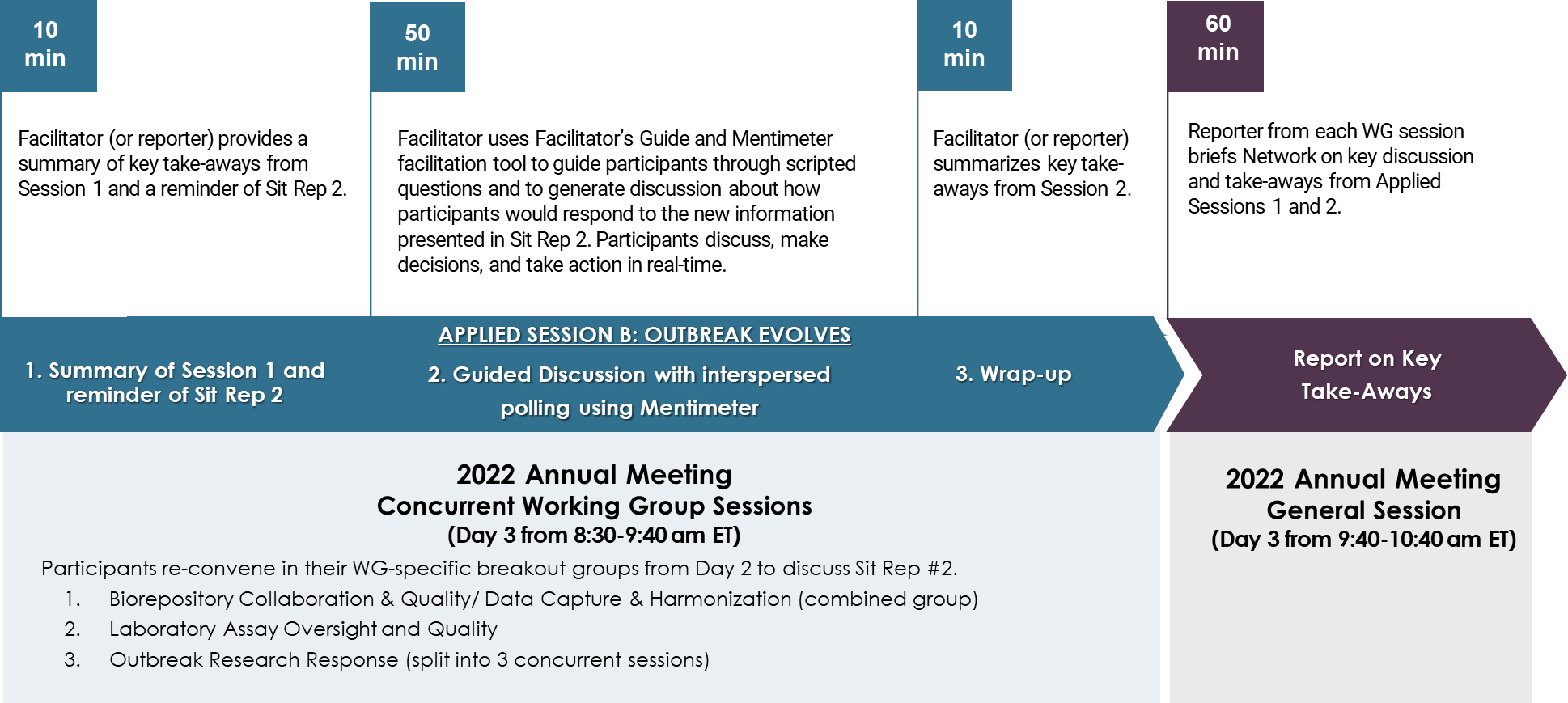 |

## TTX Scenario

The TTX focused on a fictional EID outbreak of an unknown Pathogen X of suspected animal origin (note, Pathogen X outbreak was modeled after a possible regional mpox outbreak). The scenario was presented in the format of a SitRep, simulating the type of report that is presented by the World Health Organization (WHO) or national governments to provide decision-makers with a clear and concise notification, update and understanding of a disease outbreak situation. Two complementary SitReps were developed (see **Supplemental Materials** for complete SitReps).

- **SitRep #1** described the events at the start of the outbreak. At this point in the outbreak, the pathogen was unknown, but it was suspected to be of animal origin. SitRep #1 served as the primary tool for Applied Session A – “Outbreak Identified.”
- **SitRep #2** described the outbreak’s spread and changing dynamics. Diagnostic activities have confirmed that the pathogen is of animal origin, with multiple introductions from animal reservoirs into the human population. SitRep #2 served as the primary tool for Applied Session B – “Outbreak Evolves.”

### Scenario A: Outbreak Identified

**Key Issues**

- WHO received reports of a Pathogen X-like illness in Brazil, China, Democratic Republic of Congo (DRC), France, Guinea, Senegal, Sierra Leone, and Uganda. Clustering of cases has occurred in DRC (n = 16), Guinea (n = 6), Sierra Leone (n = 21), and Uganda (n = 7). Cases in West and Central Africa, 27 reported a history of animal exposure. Isolated cases have also been identified in Brazil, China, France, and Senegal.
- All cases presented with fever and malaise. Other common symptoms include rash, swollen lymph nodes, cough, anorexia, myalgia, nausea, and skin lesions. Clinical reports from the Central Africa cluster (DRC, Uganda) suggest greater disease severity and a higher case fatality rate than other locations. A total of four deaths have been reported (2 in DRC; 1 in Sierra Leone; 1 in Uganda). One death has been linked to an immunocompromised patient. No further information is available about the other deaths.
- Preliminary evidence shows an epidemiological link between cases in Guinea and Sierra Leone and a separate link between cases in DRC and Uganda; there is no known evidence of epidemiological linkages between cases in West Africa (Guinea, Sierra Leone) and Central Africa (DRC, Uganda).

### Scenario B: Outbreak Evolves

**Key Issues**

- A total of 366 cases have been reported (63 suspected; 296 confirmed) in 15 countries globally. This accounts for an additional 7 countries (Cameroon, Liberia, Nigeria, Thailand, Germany, Ecuador, and the United States) and an increase of 303 cases since the last report. The majority of cases (58.7%) are found in the Africa region, but large clusters have also emerged in Europe and the Americas.
- More than 50% of cases in Africa report recent animal exposure, including rodents, bats, monkeys, squirrels, wild pigs, dogs and cats, and livestock (poultry, pigs, and goats). Outside of Africa, <10% of cases report a recent history of animal exposure and only 20% of cases report a recent history of travel.
- Twenty-one deaths have been reported to date, an increase of 17 since the last report. The overall case fatality rate is 5.7%, with a higher rate observed in Central Africa (11.1%).
- Fever is the most common clinical symptom among cases (91.5%), followed by malaise, rash, skin itching, skin lesions, swollen lymph nodes, headache, back pain, sore throat, cough, photophobia, joint stiffness, and difficulty breathing. Based on available data, approximately 35% of cases require hospitalization, although hospitalization rates are much higher in Africa (up to 65%).
- Preliminary laboratory investigation suggests that the unknown illness in SitRep #1 is Pathogen X and that there are two separate clades, each with a different pathology.
- Cases appear more prevalent in men than women; cases range from 1 to 77 years of age. In all regions but Africa, the most affected group is 21–30 years; in Africa, the most affected group is 10–21 years old.

## Other TTX Tools

In addition to the SitReps, a number of other TTX tools/materials were developed to ensure the effective and successful implementation of the hybrid format TTX. These included the following:

- Various MS PowerPoint slide decks outlining the TTX rationale, goals and objectives, and structure.
- A TTX flyer/insert that was included in the CREID Network 2022 Annual Meeting Welcome Packet.
- A Facilitator Handbook to guide TTX Facilitators on how to implement the TTX, including common norms and practices to successful facilitation of such an exercise (see **Supplemental Materials**).
- Preparation of a slide deck in the “[Menti](https://www.mentimeter.com/)meter” (Menti) platform, an eponymous web application allowing for real-time feedback (polling/surveying) during meetings and presentations (see **Supplemental Materials**).
- A Facilitator’s Training video, walking facilitators through the Facilitators Handbook and Menti platform for online collaboration and interaction during the TTX.

## TTX Attendance

Because of the high in-person and online attendance at the Annual Meeting, CREID Network members and NIH/NIAID observers were assigned to five breakout groups prior to the meeting, based on their CREID Network WG membership and whether they were attending the meeting in person or virtually. A total planned 359 TTX “Players” from all 10 CREID RCs were assigned to the groups, 154 and 205 of whom were expected to be in person and virtual, respectively (see **Supplemental Materials**). **Table 2** describes the composition of the assigned breakout groups. More than 150 Annual Meeting attendees participated in the applied sessions. However, the actual number of Players fluctuated across the 2 days of the exercise because of time zone conflicts or requests from Players to attend a different group because it better aligned with their role in the Network or area of expertise. Reflections of attendees with regard to the TTX’s four objectives are summarized in **Sections 4–8** of this report and are based on live discussions and answers to questions within the Menti tool during the breakout sessions.

**Table 2. Composition of Assigned TTX Breakout Groups (based on meeting registration)**

| **Working Group** | **Facilitators** | **Reporter** | **Total Players** | **# In person** | **# Virtual** | **# NIH/NIAID Observers** |
| --- | --- | --- | --- | --- | --- | --- |
| Biorepository Collaboration and Quality + Data Capture and Harmonization | Tony Moody  Nikos Vasilakis  Nathan Vandergrift  Cecelia Sanchez | Hilary Bouton-Verville | 83 | 31 | 52 | 10  3 in-person  7 virtual |
| Lab Assays | Greg Sempowski  Bob Garry | Nefer Batsuli | 65 | 23 | 42 | 8  1 in-person  7 virtual |
| Outbreak Research Response (Group A) | Peter Rabinowitz  Jay Hemingway-Foday | Megan Averill | 30 | 30 | 0 | 0 |
| Outbreak Research Response (Group B) | Richard Reithinger  Christine Johnson | Hongying Li | 70 | 70 | 0 | 15 |
| Outbreak Research Response (Group C) | Rob Breiman  Souleymane Mboup | Danielle Wagner  Aaron Macoubray | 111 | 0 | 111 | 30 |

## TTX Evaluation

Post-TTX, an evaluation of the exercise was conducted as part of the Annual Meeting’s evaluation—76% of Meeting attendees participated in the TTX, 78% of respondent strongly agreed or agreed that the TTX met its desired goals, 79% strongly agreed or agreed that the content and materials were appropriate for the exercise, and 65% strongly agreed or agreed that the duration of the exercise was appropriate (**See Supplemental Materials**).

# **Findings: Cross-Network Relationship-Building and Engagement (Objective 1)**

**Objective 1: Foster Cross-Network Relationship Building and Engagement**

Build capacity to plan and implement outbreak-related research by facilitating cross-Network knowledge-sharing, relationship-building, and collaboration.

Given the duration of the COVID-19 pandemic, the CREID 2022 Annual Meeting was for many CREID Network members the first opportunity to reconnect in person; indeed, for some attendees, the meeting represented the first time that they ever had met their RC colleagues and collaborators.

Additionally, the meeting represented an important opportunity for members of one RC to meet members from another RC: although 61% (11/18) of Menti respondents said they did not know anyone in another CREID RC present in the breakout group, after the TTX 100% (9/9) respondents felt more comfortable interacting with a CREID member from a different RC than prior to the TTX—a change in behavior that likely was facilitated by CREID members meeting and actively interacting with one or more members of other RCs/RSs during the TTX (24 respondents).

Of respondents, 84% (31/37) said that they had learned something new about another RC/RS during the TTX—their geographic presence, the scope of their research within and outside of the CREID Network, the linkages with government counterparts and other stakeholders, and general technical and operational capabilities. Thus, four respondents said that they were surprised to learn that four RCs had RSs are in Senegal, and they were marveling at the opportunities to collaborate and work together. Similarly, two respondents mentioned that they were unaware how some RCs had very close ties with country ministries of health (MOHs) and how tightly they were involved in their respective country’s public health emergency response efforts.

# **Findings: Familiarity with Network Tools and Resources (Objective 2)**

**Objective 2: Increase Familiarity with Network Tools and Resources**

Increase awareness of existing Network tools/resources.

Identify undocumented resources at RC/RS that can facilitate ORR.

Since its inception in early 2020, the CREID Network CC has developed a number of tools and resources to catalog and share RC/RS geographic presence, research capabilities, infrastructure, and needs and capacities, and provide these to RCs/RSs to successfully conduct their CREID supported research and potentially respond to outbreaks—familiarity with these tools among RCs/RSs is variable.

During the TTX, Players in the breakout groups completed an outbreak notification and support request form and were supported to navigate to and use various CREID tools and resources.

Of 58 Menti respondents, 69% (40) and 67% (39) said that they were familiar with the Network Inventory and Lab Assay Dashboard, respectively; this was followed with familiarity of the Site Capacity Dashboard (55%, 32), ORR Resources (53%, 31), Research Studies Inventory (52%, 30), RC/RS Map (47%, 27), and General Directory (40%, 23). Of respondents, only 24% (14) and 7% (4) were familiar with the CREID Resource Library and MS Teams Collaborative Space, respectively.

TTX Players mentioned additional tools and resources that the CREID Network could potentially include in an overall ORR framework: inventory of in-country partners and stakeholders that request assistance in the event of an outbreak, reagents and kits (possibly combined in a “outbreak suitcase”), support for data analytics, guidance on how CREID RCs/RSs can be officially recognized by country MOHs and other government entities, and guidance on immediate and unified communication with other stakeholders.

# **Findings: Network Readiness for ORR (Objective 3)**

**Objective 3: Assess Network Readiness for ORR**

Test assumptions about the use of Network tools/resources by Network members.

Identify and document unmet needs and knowledge gaps that may hinder research response.

When discussing SitRep#1 and SitRep#2, TTX Players discussed various aspects of network readiness/ preparedness, factors and variables that may challenge ORR efforts, and things to be strengthened or improved to ensure that ORR efforts are effective and expeditious. Generally, needs and recommendations were discussed verbally or communicated via the Menti platform. Recommended actions coalesced around two major themes: (1) Building/Leveraging Partnerships and (2) Improving CREID Network Resources.

## Building and Leveraging Partnerships

Recommendations for building or leveraging partnerships centered on two central areas, described below:

- **Within CREID-focus countries (External)**: TTX Players acknowledged that it takes time to cultivate productive relationships and establish buy-in with in-country partners for outbreak-related research, even among longstanding partnerships. As such, there was strong agreement across all groups that in-country relationship-building should be viewed as a foundational activity that begins well in advance of an outbreak. The “One Health” Ministries (i.e., Health, Agriculture/Livestock, and Environment) were consistently cited as the most important in-country partnerships. As noted by a TTX Player, building strong relationships with these ministries will help ensure that CREID researchers are “at the table when an outbreak occurs, and the response is launched.” TTX players further emphasized that an important component of relationship-building is ensuring that in-country partners are well informed of the Network’s mission and capabilities.
- **Between CREID RCs and RSs (Internal):** TTX players across all groups consistently cited the importance of ongoing efforts to build relationships within and between Network RCs and RSs. It was noted that creating these connections before an outbreak occurs will better position the Network to initiate a prompt and coordinated response when an outbreak does occur.

**Table 3** provides examples of feedback received from TTX Players about the considerations for building and leveraging external and internal partnerships.

**Table 3. Session Feedback on Building Partnerships**

| Considerations/Recommendations | Type of Partnership |
| --- | --- |
| Relevant Ministries (e.g., Health, Agriculture/Livestock, Environment) will first rely on Reference Labs, whether or not they are part of CREID. They will have access to all EID outbreak samples and will try to answer research questions with their partners. We need to make sure these ministries are informed of the CREID Network and the role it can play in advance of outbreaks. This takes time and effort, even for those RCs with longstanding in-country relationships. | External |
| Ensure that CREID Network RCs/RSs know who the key players are in terms of outbreak public health and research response in each country where Network is present, that relationships are established (specifically MOHs and outbreak “Command Center”), and that these players know of CREID offerings 🡺 ideally buy-in for CREID to work needs to be there before an outbreak occurs. | External |
| Ensure that the CREID Network is seen as genuine and trusted collaborators, strengthening the capacity of local experts, and helping them to report out on the work they are doing in response to outbreak (“telling the country’s story”). | External |
| With which MOHs, Ministry of Agriculture, National Reference Laboratories, and other stakeholders do we already have relationships to help shape research questions? | External |
| Identify local and national agencies to work with. | External |
| Engage geographers and social scientists. | External |
| Avoid overlap in US and international efforts. | External |
| Establish long-term cohorts positioned around pathogens of highest priority in focus country, but open to identifying emerging pathogens (e.g., adapting existing studies/sites with CREID-ECA in DRC). | External |
| Create connections within and between RCs and RSs BEFORE an outbreak occurs   - Know who the key points of contact are - Know if staff have changed | Internal |
| Create interest groups, lightning talks, journal clubs for cross-RC collaboration and communication, particularly for more early career researchers. | Internal |

## Improving CREID Resources

During the TTX applied sessions, Players were asked to consider what CREID resources and tools are needed to mobilize an ORR. This provided insight into familiarity with and functionality of existing resources, and resource enhancements or additions that will improve the Network’s ability to launch prompt and coordinated ORR in the future. Feedback from TTX Players for improving existing and new resources is provided in **Table 4**.

**Table 4. Feedback for Improving CREID Resources**

| Considerations/Recommendation | Type of Resource |
| --- | --- |
| Add site-wide search capability to Portal. | Existing Resource |
| Continue to familiarize ourselves and build out the CREID Network online portal for access to information and resources. | Existing Resource |
| Provide clear instructions for submitting Network Outbreak Alerts and Support Requests. | Existing Resource |
| Improve timeliness of ORR request/responses. Hold RCs responsible for responding to ORR requests. Generate reminder emails automatically. | Existing Resource |
| Continue ORR WG meetings; these are very helpful to augment searchable capabilities in Portal with real-time information and updates. | Existing Resource |
| Establish a repository of reagents, supplies materials at RSs 🡺 virtual repository. | Resource Under Development |
| “Deposit” reagents into a CREID virtual (bio)repository that you are willing to share in the CREID Network | Resource Under Development |
| Inventory key biological reagents within the network (Virtual Repository). | Resource Under Development |
| Have permit templates in place and have a central resource for permit requirements and process. | Existing and New Resource |
| Identify inherently anonymized samples that could be tested, such as wastewater surveillance samples. | New Resource |
| “Sign-up” sheets of RCs about capabilities that they can provide in given geography, pathogen, expertise 🡺 enhance and make it task specific (e.g., mpox lab assay) [Tiger Team]. | New Resource |
| Where possible, create approved Institutional Review Board (IRB) protocols in advance of an outbreak   - Share approved protocols with other IRBs, boilerplate language - Create shared protocols (inactivation protocols across the network) | New Resource |
| Create “good” practices guides for handling isolates. | New Resource |
| Do not wait until outbreak becomes a problem—we need universal assay platforms for early pathogen identification. | New Resource |
| Identify other tasks that can be done at the local level in advance of an outbreak. | General Recommendation |
| Overcome cold chain shipping concerns. | General Recommendation |
| Work with CREID sites and partners to get regional labs up to BSL-3 / US select agent level. | General Recommendation |

# **Findings****: Evidence Gaps and Research Priorities for TTX Focus Pathogen (Objective 4)**

**Objective 4: Identify Evidence Gaps and Research Priorities**

Work collectively to identify evidence gaps and research priorities for TTX pathogen X (mpox)**.**

Apply findings to plan for allocation of Network resources.

The simulated Pathogen X outbreak was based on mpox, with the goal of leveraging the TTX to facilitate development of a roadmap for Network engagement in critical research during the current global multi-country outbreak. On Day 2, TTX Players were informed that Pathogen X was mpox and were asked to consider potential evidence gaps and priorities for mpox-related research. This information could then be used by the Network for strategic planning and resource allocation. However, the 70-minute timeframe that was allocated for Applied Session B did not allow for in-depth and exhaustive discussion, as planned. Instead, these discussions will be continued as part of ongoing ORR WG activities. Because of the hybrid nature of this TTX, with Players joining from multiple time zones, and competing priorities during the Annual Meeting, longer sessions were not feasible but should be considered for future TTX activities.

Although the intended goal for this objective was not achieved, the collaborative discussions generated important general recommendations to better position the Network to engage in ORR, independent of pathogen. This includes a list of critical research questions that should be considered for all EID outbreaks (**Table 5**). This list can be used to plan for research activities that could be conducted during inter-outbreak periods or rapidly launched at the start of an outbreak, including identification of facilitating resources and collaborative partnerships both within and outside the Network.

**Table 5. Critical Research Questions Relevant to any EID Outbreak**

| Epidemiology |
| --- |
| What are the ecological factors driving the outbreak? |
| What type of granular geospatial information can help better define outbreak dynamics? Relationships between location, environmental factors, and health outcomes; Geospatial mapping; Geographic spread. |
| Are there common species? What species are people interacting with? |
| What is the animal ecology relative to outbreaks (e.g., seasonality)? |
| What is the asymptomatic infection rate? |
| What are the population demographics? |
| How can we use predictive modeling to understand where the outbreak will go next? |
| What is the temporal nature of the outbreak? |
| What is the seroprevalence? |
| What are the bushmeat supply routes and transportation networks? |
| Genetics |
| Phylogeographic data, R_0_ ? |
| What is the sequence of the pathogen? |
| What are host genetic factors? |
| What are differences in clinical phenotypes? |
| What is pathogenicity of different clades? |
| What is needed to define clades and sequence variation? |
| Are there other surveillance data that we could tap into (e.g., wastewater)? |
| What are the viral sequalae and long-term effects? |

| Host Dynamics |
| --- |
| What is the animal reservoir? |
| Is the infection rate increasing in the animal reservoir? |
| How has the animal/host range of the virus changed? Is there a functional genomic change at play? |
| What is the origin of the pathogen and when/how did spillover into animals/humans occur? |
| What are the host factors/comorbidities? |
| Transmission Dynamics |
| What is the mode of transmission and course of infection? |
| What modes of transmission can we predict based on other outbreaks? |
| Symptomatic vs. asymptomatic infection (viral load/transmission)? |
| What are the transmission dynamics in both human and animal cohorts? |
| What role does long-term immunity play? |
| Is transmission zoonotic or human-to-human, or both? |
| Diagnostics |
| What are best ways to detect the cases accurately? |
| What are appropriate sample-collection procedures? |
| What is the specificity of available tests? Is there any cross-reactivity with other pathogens? |
| Therapeutics |
| What antiviral drug screening exists? |
| What antivirals, other therapeutics, repurpose licensed vaccines are needed? (*Note: CREID can fund mAbs for translational research.)* |
| Discovery and design of therapeutic antibodies? |
| Data/Analytics |
| What is the Metadata structure? |
| What data would be needed for predictive modeling? |
| What is the analysis capacity of sequence data onsite? |

## Other Considerations for ORR

Several other important considerations were noted during the discussion of mpox-related research needs—again, these are applicable to all future outbreaks and are presented below.

- Animal expertise is critical for any ORR effort of a pathogen of zoonotic origin, including animal sampling/engagement of veterinarians.
- It is important to understand the social science/anthropology of outbreaks and engage local communities.
- Clinical research can help complement/complete the full picture of an outbreak.
- It is important to understand the dynamics, politics, and sensitivities around collaboration with other researchers or research networks, including sometimes other competing networks.
- The Network should consider how much information and data can be shared by the larger CREID Network while remaining sensitive to local and national government efforts.
- Collaboration among RCs is essential and should be established before an outbreak occurs.
- The Network should ensure that there is enough reference material for development and validation of tests/protocols (no commercial assays/everything is in house).
- The Network should leverage existing resources to optimize research capacity, including:
  - Ongoing cohorts at RCs/RSs or in CREID focus countries that are already in place
  - Integration with natural history collections
  - In-country diagnostic and sequencing capacity and supplies
  - Documentation of available samples so that they can be quickly accessed when needed

# **Recommendations**

## Objective 1: Cross-Network Relationship-Building and Engagement

- The CREID Annual Meeting represents an important venue for CREID Network members (i.e., RC and RS colleagues/collaborators) to meet and connect, foster new collaborative linkages, incubate ideas, and learn about the Network and the resources it provides.

***Recommendations:***

- *Continue to ensure that a CREID Annual Meeting is held (domestically or Internationally), as it represents a crucial platform for CREID RCs/RSs to meet and connect, foster new collaborative linkages, incubate ideas, and learn about the Network and the resources it provides.*
- *Ensure that the Annual Meeting’s agenda includes not only research activities the RCs are conducting with CREID support, but also non-CREID collaborative research, an overview of infrastructure and capabilities, and in-country linkages with stakeholders such as MOHs—this would allow other RCs/RSs to understand the true potential and added value of any given RC/RS, and potential scope of collaborative linkages.*

## Objective 2: Familiarity with Network Tools and Resources

- The CREID CC has developed various tools and resources to catalog and share RC/RS geographic presence, research work, infrastructure, and needs and capacities, and provide these to RCs/RSs to successfully conduct their CREID-supported research and potentially respond to outbreaks—familiarity is variable.

***Recommendations:***

- *Although some of the tools and resources the CREID CC has developed are known to and used by RCs/RSs, effort should be made to increase the RCs’/RSs’ knowledge and use even more—this could include highlighting the tools and resources during SC, WG, and Community of Practice meetings, in the CREID newsletter, or through other dissemination channels.*
- *A review should take place into why some tools and resources are more known than others; for those that are little or unknown to RCs/RSs, a discussion should be held whether the tools and resources are crucial to the Network’s success and what approaches could be adopted to improve RCs’/RSs’ knowledge and use of them, or whether they should be discontinued.*
- *The MS Teams Collaborative Space is not known to and used by RCs/RSs—separate login credentials being the main challenge. Network tools and resources should be migrated off Teams to the secure private side of the CREID Network website.*
- RCs/RSs mentioned a number of additional tools and resources the Network could consider adding if team effort and resources allow.

***Recommendation:***

- *CREID WGs/CoPs, DMID, and the CC should discuss the value of adding the following tools and resources: country institutional/stakeholder mapping briefs for every country CREID is supporting research in; branding and communication guidance for how RCs can engage with MOHs and other government entities as part of a revised and simplified ORR framework; and virtual specimen/reagent (bio)repositories.*

## Objective 3: Assess Network Readiness for ORR

- Various aspects of network readiness/preparedness, what factors and variables may challenge ORR efforts, and what needs to be strengthened or improved to ensure that ORR efforts are effective and expeditious were discussed—recommended actions coalesced around two major themes: (1) Building/Leveraging Partnerships and (2) Improving CREID Resources.

***Recommendations for Building/Leveraging Partnerships:***

- *In any country where CREID is supporting research, RCs/RSs should establish linkages and relationships with MOHs and other government entities (e.g., ministries of agriculture and livestock, ministries of education), and other key multilateral, bilateral, nongovernmental, and academic stakeholders. This will not only help them in the current research (e.g., in being included in the decision-making as part of an overarching public health response, or in terms of IRB/ethics approvals), but particularly in the event of an outbreak and possible ORR. Note, although the CREID institutional/stakeholder mapping briefs can educate RCs/RSs on a given country’s EID landscape, they will have to invest local capital to establishing these linkages and relationships.*
- *RCs/RSs should create connections within and between prior to an outbreak occurring so that resources can be mobilized when the outbreak does occur—knowing RCs/RSs infrastructure and operational/staffing capabilities, and in-country linkages with stakeholders such as MOHs and other stakeholders (see above) are critical to effectively and expediently launching research activities in the event of an outbreak. Other approaches could include interest groups, lightning talks, or journal clubs, either as part of established CREID WG/Communities of Practice and Scientific/SC meeting series, or outside of these.*
- *As much as possible RCs/RSs should build an intra/inter RC/RS roster of experts who can respond to the whole scope of any given ORR, including geographers, social scientists, and anthropologists.*
- *In the event of an outbreak, clear roles and responsibilities of the RCs/RSs engaged in the ORR effort should be defined at the outset, to avoid overlapping efforts, misrepresentation, and miscommunication.*

***Recommendations for Improving CREID Network Resources:***

- *ORR WG meetings are seen as valuable for information sharing and ORR relevant updates, and highlighting CREID tools and resources. Independently, RCs/RSs should continue to familiarize themselves with the CREID private portal and available tools and resources.*
- *When outbreaks of priority pathogens or pathogens of interest do occur in a country where RCs/RSs are present, RCs/RSs should provide the necessary information for ORR outbreak alert notifications and support requests to be completed by the CREID CC.*
- *Any outbreak in a country where CREID is supporting RCs/RSs to conduct research should result in rapid response team (Tiger Team) to conduct the ORR—ideally, this would be led by the RS(s) in that country; other RCs/RSs could, depending on capabilities and priorities, be part of that team.*
- *Consider adding a general site-wide free-text search capability to the CREID Network website (public and private). This could allow RCs/RSs to retrieve information or locate relevant site dashboard tools, which becomes increasingly important as new resources and tools are added. Improving the user experience will help foster website engagement and make it easier for RCs/RSs to familiarize themselves with available resources and tools.*
- *The CREID Network private portal should include a virtual repository of biospecimens, reagents, pathogens isolates, and materials at any given RS. Alternatively, RCs/RSs should specify exactly what reagents and materials they need when providing the CC with the information to complete the outbreak alert notification and support request form.*
- *CREID’s Resource Library should include standard/template “boilerplate” human study protocols and other relevant documents (e.g., good practice guides for collecting samples and handling isolates, inactivation protocols)—this would facilitate expediently mobilizing an ORR effort as RCs/RSs could submit study protocols to country IRBs prior to outbreaks occurring and RC/RSs would have IRB approvals, protocols, and other guidance in place for when an outbreak does occur.*
- *Discussions should take place with DMID on how CREID RSs can build the country or regional infrastructure to handle samples/pathogens at BSL-3 and 4/US select agent level—this would ensure that the ORR effort is more effective, local, and does not have to rely on US-based infrastructure and capabilities.*
- *Discussions should take place with DMID on how to improve RSs cold chain shipping capabilities in-country.*
- *Discussions should take place with DMID and RCs/RSs on what type and how long-term cohorts of susceptible human and animal populations could be established, so that these are readily available for sampling in the event of a priority pathogen or pathogen of interest outbreak.*
- *CREID RC and RS investigators need to embrace the NIH/NIAID requirement for public sharing of data assets associated with CREID-funded publications and shared protocols.*

## Objective 4: Evidence Gaps and Research Priorities for TTX Focus Pathogen

- The TTX provided an opportunity for real-time, collaborative discussions about mpox evidence gaps and research priorities. However, because of TTX time constraints and the nature of the global multicountry mpox outbreak at the time of the TTX activity, these discussions were not as beneficial as envisioned. Nonetheless, important general recommendations were identified to better position the Network for future engagement in any EID ORR, independent of pathogen. These recommendations, as described in **Section 7**, will be considered for strategic planning of CREID Network research priorities, resource allocation, and collaboration-building.
- There was a consensus to engage Network laboratories to develop multiplex assays to distinguish mpox from other orthopox viruses. Note, this activity proceeded with two RCs being actively engaged.

# **Implementation Plan**

The TTX accomplished its primary objective of stress-testing the CREID ORR framework 2.5 years into the 5-year award to develop and stand up this innovative global outbreak research network. The recommendations generated by the 2-day TTX lay the groundwork for the development of a revised and simplified CREID Network ORR Framework and the development/augmentation of Network tools and resources.

The revised ORR Framework, to be finalized mid-2023, will help the Network achieve its goal of timely, effective, coordinated, and responsive outbreak-related research by:

- Updating and expanding the tools and resources the CREID CC is curating and making available to RCs/RSs, including institutional mapping country briefs to help RCs/RSs navigate the in-country stakeholder landscape and establish relationships with key actors prior to an EID outbreak occurring; standard/template human subject protocols and other relevant documents; a Network pathogen surveillance dashboard/map tool; and a virtual specimen/reagent (bio)repository.
- Providing consistent guidance to the Network in terms of investigator (RC/RS), DMID, CC, and stakeholder roles and responsibilities during an ORR, CREID Network representation in country, and the availability of existing and new tools and resources (e.g., on branding and media communication, on in-country government and nongovernment stakeholder engaged in outbreak public health emergency and research response).
- Establishing a focused multidisciplinary CREID Tiger Team when an outbreak of any priority pathogen or pathogen of interest occurs, with representation from the in-country RCs/RSs, DMID, CC, key stakeholders, and RCs/RSs with needed capabilities and expertise.

The revised CREID Network ORR Framework will help facilitate Network engagement with critical external collaborators and improve central coordination within the Network by providing mechanisms to leverage cross-RC or cross-RS synergies, avoid duplication of efforts, and conduct impactful research in response to outbreaks of priority pathogens and pathogens-of-interest.
